# Supplementary material for: Variation in appropriate diabetes care and treatment targets in urban and rural areas in England: an observational study of the ‘rule of halves’
Source: BMJ Open. 2022 Feb 16;12(2):e057244. doi: 10.1136/bmjopen-2021-057244 (PMC8852726; doi:10.1136/bmjopen-2021-057244)
Supplement: Supplementary data [file bmjopen-2021-057244supp001.pdf]

SUPPLEMENTARY MATERIAL

DATA

Data on urbanity

The 2011 rural-urban classification (RUC) of clinical commissioning groups (CCG) in England is based on the 2011 RUC of underlying output areas (small-level geographical units). It classifies CCGs into seven classes of the RUC based on the proportion of its population in underlying output areas residing in rural settings. There is also a broader hierarchical grouping of three classes of the RUC which groups CCGs into the following: predominantly rural (at least 50% of the population living in rural settings); predominantly urban (less than 25% of the population living in rural settings; and urban with significant rural (between 25% and 50% of the population living in rural settings).

NDA

Data are extracted from both general practice clinical systems and specialist units in secondary care hospitals (24). The audit is voluntary for general practices and in 2016-17 the participation rate was 95.3 per cent of all practices in England and Wales. For specialist units in secondary care, participation in the audit is compulsory. Whilst the data used in this study are nationally published practice-level data, they are aggregated from individual-level identifiable data from which patients can opt-out.

The 2016-17 NDA covers the majority of England (and Wales) with a participation rate of 95.3% (24). Information is collated from GP clinical systems and comparable data are collected from secondary care providers over a six week period. Data on registrations represent counts of patients who have a recorded diagnosis of diabetes in these data returns.

METHODS

Estimating mean rates of annual prevalence from the UKHLS

In each wave of the UKHLS, participants are asked various questions about their general health – including questions about whether they have any of a specific set of health conditions including diabetes. The participant then gives information on which conditions apply to them (if any) from the specified list. In addition to these questions about health which are asked in each wave, in waves 2 and 3 (2010-2012) respondents had a nurse health assessment in which a range of biomedical measures were collected including glycated haemoglobin (HbA1c) measurement which is an indicator of diabetes.

We combined these variables to create an indicator of whether an individual had diabetes or not in each wave of the UKHLS, and retained data for wave 7 to align with the time period covered by other data sources in this study.

RESULTS

The levels of the ROH in urban and rural CCGs

Estimated prevalence of diabetes

From the UKHLS we estimated the overall prevalence of diabetes at wave 7 (2015-17) in England as 7.69% (95% CI [7.25%; 81.3%]) (Supplementary Table S1). For those residing in urban settings this was 7.80% (95% CI [7.30%; 8.31%]), compared with 7.24% (95% CI [6.32%; 8.16%]) for those residing in rural settings (Table 2; Supplementary Table S1).

Supplementary Table S1: Estimates of diabetes prevalence for England (2015-17) derived from UKHLS

	N	Mean	95% CI
Overall	33,336	7.69%	[7.25%; 8.13%]
Predominantly Urban	27,374	7.80%	[7.30%; 8.31%]
Predominantly Rural	5,962	7.24%	[6.32%; 8.16%]

Notes: estimated from UKHLS Wave 7 (2015-2017), longitudinal sample weights applied

Diabetes registrations in the NDA

Predominantly urban CCGs had on average 14,750 people registered as having diabetes ((14,750/21,206); 69.55% of estimated prevalence). This compared to an average of 14,089 in predominantly rural CCGs ((14,089/19,665); 71.64% of estimated prevalence) (Supplementary Table S2).

Provision of appropriate diabetes care quality

On average 6,763 of 14,750 registered people with diabetes in predominantly urban CCGs received all eight appropriate care processes (45.85%) (Supplementary Table S2). In rural settings, this figure was 6,948 of 41,089 registered with diabetes (49.32%).

Achievement of diabetes treatment targets

On average, 5,267 of the 6,763 patients receiving appropriate care achieve diabetes treatment targets in predominantly urban CCGs (77.88%) (Supplementary Table S2). This compares to 4,934 of 6,948 in rural settings (71.01%)

Mason T, *et al. BMJ Open* 2022; 12:e057244. doi: 10.1136/bmjopen-2021-057244

Supplementary Table S2: Descriptive Statistics for CCGs

		Predominantly Urban CCGs (N=121)		Predominantly Rural CCGs (N=32)	
**Population size and age/sex structure**					
**Variable**		Mean	SD	Mean	SD
Population size		283,640	166,226	285,284	178,929
% Under 40		52.85%	6.47%	43.64%	3.34%
% 40-64		31.25%	2.78%	33.84%	1.18%
% 65-79		11.62%	2.95%	16.56%	2.00%
% 80+		4.28%	1.16%	5.97%	0.74%
% female		50.49%	0.86%	50.72%	0.51%
**Diabetes prevalence, registrations, appropriate care and treatment targets**					
**Variable**		Mean		Mean	
NDA participation of general practices		95.84%		93.83%	
Participation adjusted population size^		271,716		270,914	
Estimated prevalence	Total (in adj. population)**	21,206 [19,847; 22,566]		19,665 [17,115; 22,098]	
	% of population*	7.80% [7.30%; 8.31%]		7.24% [6.32%; 8.16%]	
Registrations	Total	14,750		14,089	
	% of est. prevalence	69.55%		71.64%	
Appropriate care	Total	6,763		6,948	
	% of est. prevalence	31.89%		35.33%	
	% of registrations	45.85%		49.32%	
Treatment targets	Total	5,267		4,934	
	% of est. prevalence	24.84%		25.09%	
	% of registrations	35.71%		35.02%	
	% of appropriate care	77.88%		71.01%	

Notes: *prevalence data estimated from UKHLS Wave 7 (2015-2017), 95% CI in parentheses; ** Total derived from prevalence rates from the UKHLS and population and rurality data from ONS; registrations, quality and treatment from NDA

Practice population characteristics and workforce supply

Supplementary Table S3 summarises the data on practice population characteristics and workforce supply for predominantly urban and rural CCGs.

Practices in predominantly urban CCGs have 46.02% of those with a diabetes record in receipt of all eight care processes, compared with 49.39% for practices in predominantly rural CCGs (Supplementary Table S3). Practices in predominantly urban CCGs have 46.02% of those with a diabetes record in receipt of all eight care processes, compared with 49.39% for practices in predominantly rural CCGs. Practices in rural CCGs serve older diabetic patients – 45.64% are 65 and older compared with 36.53% in urban settings (Supplementary Table S3).

Practices in predominantly urban CCGs have more of their diabetic patients residing in the most deprived 40% of neighbourhoods (54.2% compared with 31.09% in rural CCGs). Practices in predominantly rural CCGs have a much higher share of diabetic patients (94.99%) with known ethnicity recorded as white compared with those in predominantly urban CCGs (70.54%) (Supplementary Table S3).

Finally, practices in predominantly rural CCGs have more FTE GPs, nurses and administrative staff per 1,000 patients compared with urban CCGs. This is most pronounced in terms of nurses (0.39 per 1,000 for urban vs. 0.51 per 1,000 for rural), 33.15% more – but is still notably bigger in terms of GPs (9.7% more).

Supplementary Table S3: Care quality, treatment target achievement and practice-level indicators of need and supply in urban and rural areas in England 2016-17

	Predominantly Urban (CCGs=121)	Predominantly Rural (CCGs=32)
Number of General Practices	4,011	902
**Mean Care Quality and Treatment Target Achievement**		
% receiving all care processes	46.02%	49.39%
% achieving all three treatment targets	35.76%	35.01%
**Mean characteristics of registered diabetes and wider practice population**		
Total patients (list size)	7,603	8,931
Total registered diabetics	576	674
Practice prevalence of diabetes (%)	7.57%	7.55%
% registered diabetics male	55.50%	56.71%
% registered diabetics under 40	7.61%	5.94%
% registered diabetics aged 40-64	45.11%	37.72%
% registered diabetics aged 65-79	34.43%	40.17%
% registered diabetics aged 80+	12.10%	15.47%
% registered diabetics from most deprived 40% of neighbourhoods	54.20%	31.09%
% of diabetics with ethnicity unknown	13.01%	23.05%
% of diabetics with known ethnicity = white	70.54%	94.99%
% of diabetics with known ethnicity = minority	29.46%	5.01%
**Mean practice measures of labour supply**		
GPs per 1,000 patients	0.68	0.75
Nurses per 1,000 patients	0.39	0.51
Administrative staff per 1,000 patients	1.63	1.81

Notes: data on diabetes taken from the NDA; data on practice list size and labour supply from GP workforce data from NHS England; data reflect those practices on which complete data exist from both sources

Extension to include 'urban with significant rural' CCGs

Supplementary Table S4: National levels of the ROH by urban/rural status (including “urban with significant rural” CCGs)

	Predominantly Urban CCGs (N=121)			Predominantly Rural CCGs (N=32)			Urban with Significant Rural CCGs (N=38)		
	Estimated total population* = 32,877,630			Estimated total population* = 8,699,248			Estimated total population* = 10,914,316		
	Total	% of previous level**	% of prevalence	Total	% of previous level**	% of prevalence	Total^	% of previous level**	% of prevalence
Diabetes prevalence	2,566,014	7.80%	100.00%	627,431	7.24%	100.00%	828,615	7.59%	100.00%
Registrations	1,784,715	69.55%	69.55%	450,850	71.86%	71.86%	567,400	68.48%	68.48%
Appropriate Care Quality	818,300	45.85%	31.89%	222,345	49.32%	35.44%	273,205	48.15%	32.97%
Achieve Treatment Targets	637,325	77.88%	24.84%	157,880	71.01%	25.16%	195,750	71.65%	23.62%

Notes: *Total population adjusted for practice participation in NDA; diabetes prevalence estimated using UKHLS data for Wave 7 (2015-17); **previous level refers to row above (as denominator), prevalence refers to as % of estimated total adjusted population; ^prevalence estimate for urban with significant rural based on weighted mean of prevalence estimates for urban/rural respondents to the UKHLS (no directly comparable group in UKHLS), weights based on assumption of RUC category mid-point (37.5%) of population being rurally located

Mason T, *et al. BMJ Open* 2022; 12:e057244. doi: 10.1136/bmjopen-2021-057244

Supplementary Table S5: Descriptive Statistics for CCGs (including "urban with significant rural" CCGs)

		**Predominantly Urban CCGs (N=121)**		**Predominantly Rural CCGs (N=32)**		**Predominantly Rural CCGs (N=32)**	
**Population size and age/sex structure**							
**Variable**		Mean	SD	Mean	SD	Mean	SD
Population size		283,640	166,226	285,284	178,929	311,014	223,137
% Under 40		52.85%	6.47%	43.64%	3.34%	45.15%	2.91%
% 40-64		31.25%	2.78%	33.84%	1.18%	33.43%	1.26%
% 65-79		11.62%	2.95%	16.56%	2.00%	15.66%	1.73%
% 80+		4.28%	1.16%	5.97%	0.74%	5.76%	0.91%
% female		50.49%	0.86%	50.72%	0.51%	49.05%	0.42%
**Diabetes prevalence, registrations, appropriate care and treatment targets**							
**Variable**		Mean		Mean		Mean	
NDA participation of general practices		95.84%		93.83%		92.67%	
Participation adjusted population size^		271,716		270,914		288,217	
Estimated prevalence*	Total (in adj. population)	21,206 [19,847; 22,566]		19,665 [17,115; 22,098]		21,876	
	% of population	7.80% [7.30%; 8.31%]		7.24% [6.32%; 8.16%]		7.59%	
Registrations	Total	14,750		14,089		14,932	
	% of est. prevalence	69.55%		71.64%		68.26%	
Appropriate Care	Total	6,763		6,948		7,190	
	% of est. prevalence	31.89%		35.33%		32.87%	
	% of registrations	45.85%		49.32%		48.15%	
Treatment targets	Total	5,267		4,934		5,151	
	% of est. prevalence	24.84%		25.09%		23.55%	
	% of registrations	35.71%		35.02%		34.50%	
	% of appropriate care	77.88%		71.01%		71.65%	

Notes: *prevalence data estimated from UKHLS Wave 7 (2015-2017), 95% CI in parentheses; population and rurality data from ONS; registrations, quality and treatment from NDA

Mason T, *et al. BMJ Open* 2022; 12:e057244. doi: 10.1136/bmjopen-2021-057244

Supplementary Table S6: Care quality, treatment target achievement and practice-level indicators of need and supply in urban and rural areas in England 2016-17 (including "urban with significant rural" CCGs)

	Predominantly Urban (CCGs=121)	Predominantly Rural (CCGs=32)	Urban with Significant Rural (CCGs=38)
Number of General Practices	4,011	902	1131
**Mean Care Quality and Treatment Target Achievement**			
% receiving all care processes	46.02%	49.39%	48.29%
% achieving all three treatment targets	35.76%	35.01%	34.58%
**Mean characteristics of registered diabetes and wider practice population**			
Total patients (list size)	7,603	8,931	8,815
Total registered diabetics	576	674	656
Practice prevalence of diabetes (%)	7.57%	7.55%	7.44%
% registered diabetics male	55.50%	56.71%	56.56%
% registered diabetics under 40	7.61%	5.94%	6.14%
% registered diabetics aged 40-64	45.11%	37.72%	37.98%
% registered diabetics aged 65-79	34.43%	40.17%	39.62%
% registered diabetics aged 80+	12.10%	15.47%	15.51%
% registered diabetics from most deprived 40% of neighbourhoods	54.20%	31.09%	31.45%
% of diabetics with ethnicity unknown	13.01%	23.05%	23.51%
% of diabetics with known ethnicity = white	70.54%	94.99%	72.04%
% of diabetics with known ethnicity = minority	29.46%	5.01%	5.09%
**Mean practice measures of labour supply**			
GPs per 1,000 patients	0.68	0.75	0.69
Nurses per 1,000 patients	0.39	0.51	0.46
Administrative staff per 1,000 patients	1.63	1.81	1.74

Notes: data on diabetes taken from the NDA; data on practice list size and labour supply from GP workforce data from NHS England; data reflect those practices on which complete data exist from both sources

Mason T, *et al. BMJ Open* 2022; 12:e057244. doi: 10.1136/bmjopen-2021-057244

Supplementary Table S7: Regression estimates - analyses of the % of patients receiving appropriate diabetes care quality in general practices

	I	II	III
**Urban / Rural status (reference = predominantly rural)**			
Predominantly Urban = 1	-3.370***	-1.332	-0.914
Urban with sig. rural = 1	-1.106	-0.9	-0.776
**Practice diabetic population characteristics**			
Registered diabetics (< 250) = 1		-3.250***	-3.532***
Registered diabetics (500-749) = 1		1.698*	1.887**
Registered diabetics (750 +) = 1		2.427*	2.556*
% of diabetics aged under 40		0.264*	0.284*
% of diabetics aged 40 - 64		-0.09	-0.087
% of diabetics aged 65 - 79		0.231*	0.244*
% of diabetics = male		0.003	0.018
% of patients from most deprived 40% neighbourhoods		0.034**	0.033**
% of patients from ethnic minorities		-0.067***	-0.065***
% of patients of unknown ethnicity		-0.057***	-0.057***
List size (1,000s)		-0.02	-0.479***
**Practice labour supply**			
GPs per 1000 patients			0.443***
Nurses per 1000 patients			0.330*
Admin staff per 1000 patients			0.019
Constant	49.392***	45.199***	45.831***
R-sq.	0.005	0.042	0.048
N	6,044	6,044	6,044

* p<0.05; ** p<0.01; *** p<0.001; Model I = Urban indicator only; Model II = I + Practice population characteristics; Model III = II + Practice labour supply; Models II and III include indicators for the age structure of practice list (by sex); all models estimated at practice-level using OLS weighted by the number of registered diabetics; all models clustered by general practice

Mason T, *et al. BMJ Open* 2022; 12:e057244. doi: 10.1136/bmjopen-2021-057244

Supplementary Table S8: Regression estimates - analyses of the % of (registered) diabetic patients achieving diabetes treatment targets in general practices (including "urban with significant rural" CCGs)

	I	II	III	IV
**Urban / rural status (reference = predominantly rural)**				
Predominantly Urban = 1	0.755**	1.062***	1.259***	1.221***
Urban with sig. rural = 1	-0.43	-0.329	-0.267	-0.279
**Appropriate care quality**				
% of patients receiving appropriate care		0.091***	0.091***	0.091***
**Practice diabetic population characteristics**				
Registered diabetics (< 250) = 1			1.552***	1.582***
Registered diabetics (500-749) = 1			-0.562*	-0.583*
Registered diabetics (750 +) = 1			-0.317	-0.33
% of diabetics aged under 40			-0.270***	-0.272***
% of diabetics aged 40 - 64			0.032	0.032
% of diabetics aged 65 - 79			0.259***	0.258***
% of diabetics = male			-0.028	-0.029
% of patients from most deprived 40% neighbourhoods			-0.010*	-0.010*
% of patients from ethnic minorities			0.030***	0.030***
% of patients of unknown ethnicity			-0.026***	-0.026***
List size (1,000s)			-0.098**	-0.055
**Practice labour supply**				
GPs per 1000 patients				-0.036
Nurses per 1000 patients				-0.029
Admin staff per 1000 patients				-0.005
Constant	35.005***	30.505***	37.728***	4.872
R-sq.	0.005	0.062	0.139	0.139
N	6,044	6,044	6,044	6,044

* p<0.05; ** p<0.01; *** p<0.001; Model I = Urban indicator only; Model II = I + Care quality; Model III = II + Practice population characteristics; Model IV = III + Practice labour supply; Models III + IV include indicators for the age structure of practice list (by sex); all models estimated at practice-level using OLS weighted by the number of registered diabetics; all models clustered by general practice

Mason T, *et al. BMJ Open* 2022; 12:e057244. doi: 10.1136/bmjopen-2021-057244

[EMBED SUPPLEMENTARY FIGURE S1 HERE]
