# Supplementary figures and images for: Variation in appropriate diabetes care and treatment targets in urban and rural areas in England: an observational study of the ‘rule of halves’
Source: BMJ Open. 2022 Feb 16;12(2):e057244. doi: 10.1136/bmjopen-2021-057244 (PMC8852726; doi:10.1136/bmjopen-2021-057244)

Supplementary Figure S1: ROH for 'Urban with Significant Rural' Areas

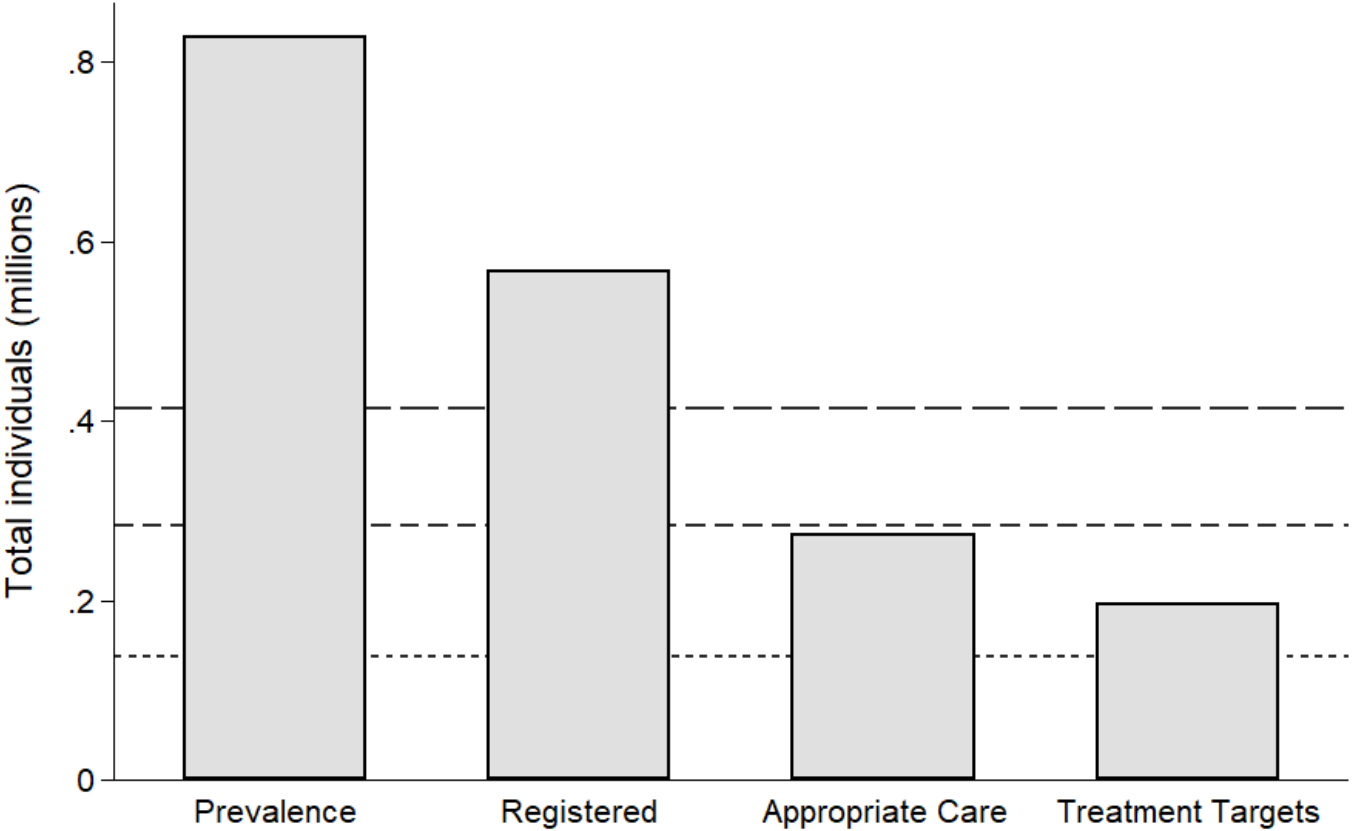

Supplement: Supplementary data [file bmjopen-2021-057244supp002.pdf]
