# Supplementary material for: Variation in appropriate diabetes care and treatment targets in urban and rural areas in England: an observational study of the ‘rule of halves’
Source: BMJ Open. 2022 Feb 16;12(2):e057244. doi: 10.1136/bmjopen-2021-057244 (PMC8852726; doi:10.1136/bmjopen-2021-057244)
Supplement: Author's manuscript [file bmjopen-2021-057244.draft_revisions.pdf]

BMJ Open

BMJ Open is committed to open peer review. As part of this commitment we make the peer review history of every article we publish publicly available.

When an article is published we post the peer reviewers' comments and the authors' responses online. We also post the versions of the paper that were used during peer review. These are the versions that the peer review comments apply to.

The versions of the paper that follow are the versions that were submitted during the peer review process. They are not the versions of record or the final published versions. They should not be cited or distributed as the published version of this manuscript.

BMJ Open is an open access journal and the full, final, typeset and author-corrected version of record of the manuscript is available on our site with no access controls, subscription charges or pay-per-view fees (http://bmjopen.bmj.com).

If you have any questions on BMJ Open's open peer review process please email info.bmjopen@bmj.com

BMJ Open

Variation in appropriate diabetes care and treatment targets in urban and rural areas in England: an observational study of the 'rule of halves'

Journal:	*BMJ Open*
Manuscript ID	bmjopen-2021-057244
Article Type:	Original research
Date Submitted by the Author:	16-Sep-2021
Complete List of Authors:	Mason, Thomas; The University of Manchester Faculty of Biology Medicine and Health<br>Whittaker, William; The University of Manchester Faculty of Biology Medicine and Health, Faculty of Biology, Medicine and Health<br>Dumville, Jo C.; The University of Manchester Faculty of Biology Medicine and Health<br>Bower, Peter; The University of Manchester Faculty of Biology Medicine and Health
Keywords:	Epidemiology < TROPICAL MEDICINE, General diabetes < DIABETES & ENDOCRINOLOGY, Organisation of health services < HEALTH SERVICES ADMINISTRATION & MANAGEMENT, Quality in health care < HEALTH SERVICES ADMINISTRATION & MANAGEMENT

SCHOLARONE™
Manuscripts

I, the Submitting Author has the right to grant and does grant on behalf of all authors of the Work (as defined in the below author licence), an exclusive licence and/or a non-exclusive licence for contributions from authors who are: i) UK Crown employees; ii) where BMJ has agreed a CC-BY licence shall apply, and/or iii) in accordance with the terms applicable for US Federal Government officers or employees acting as part of their official duties; on a worldwide, perpetual, irrevocable, royalty-free basis to BMJ Publishing Group Ltd ("BMJ") its licensees and where the relevant Journal is co-owned by BMJ to the co-owners of the Journal, to publish the Work in this journal and any other BMJ products and to exploit all rights, as set out in our licence.

The Submitting Author accepts and understands that any supply made under these terms is made by BMJ to the Submitting Author unless you are acting as an employee on behalf of your employer or a postgraduate student of an affiliated institution which is paying any applicable article publishing charge ("APC") for Open Access articles. Where the Submitting Author wishes to make the Work available on an Open Access basis (and intends to pay the relevant APC), the terms of reuse of such Open Access shall be governed by a Creative Commons licence – details of these licences and which Creative Commons licence will apply to this Work are set out in our licence referred to above.

Other than as permitted in any relevant BMJ Author's Self Archiving Policies, I confirm this Work has not been accepted for publication elsewhere, is not being considered for publication elsewhere and does not duplicate material already published. I confirm all authors consent to publication of this Work and authorise the granting of this licence.

For peer review only

Variation in appropriate diabetes care and treatment targets in urban and rural areas in England: an observational study of the 'rule of halves'

Thomas Mason[1], Will Whittaker[1], Jo Dumville[1], Peter Bower[1]

[1]*School of Health Sciences; Faculty of Biology, Medicine and Health, University of Manchester, Oxford Road, Manchester, M13 9PL*

Corresponding Author: Thomas Mason

Author email: thomas.mason@manchester.ac.uk; william.whittaker@manchester.ac.uk; jo.dumville@manchester.ac.uk; peter.bower@manchester.ac.uk

The authors of this study have no competing interest to declare

This study did not require ethical approval as it used retrospective, anonymised observational data and all results are reported at an aggregate level

MeSH keywords: diabetes mellitus; urban health; epidemiology; workforce; small-area analysis

Word count (excluding tables, figures and supplement) = 3,409

This research was supported by the National Institute for Health Research Applied Research Collaboration Greater Manchester. The views expressed in this publication are those of the author(s) and not necessarily those of the National Institute for Health Research or the Department of Health and Social Care. This research originates from work undertaken in the

For peer review only - http://bmjopen.bmj.com/site/about/guidelines.xhtml

Cities Changing Diabetes (CCD) programme for Greater Manchester in partnership with and supported by Health Innovation Manchester and Novo Nordisk. CCD was launched in 2014 by the Steno Diabetes Center Copenhagen, University College London, and Novo Nordisk.

Data sharing statement: the population and practice-level data from this study are publicly available from the Office for National Statistics and NHS Digital, the data used to generate prevalence estimates from UK Household Longitudinal Survey can be applied for via the UK Data Service.

For peer review only

ABSTRACT

Objectives

To estimate the 'rule of halves' for diabetes care for 2016-17 in urban and rural areas in England; and to examine the extent to which any differences in urban and rural settings are explained by population characteristics and general practice workforce supply

Design

A retrospective observational study

Setting

Populations resident in predominantly urban and rural areas in England (2015-17)

Participants

Respondents to the UK Household Longitudinal Survey; general practices in England reporting to the National Diabetes Audit and providing workforce data to NHS Digital

Outcomes

Diabetes prevalence; administrative records of diagnoses of diabetes; provision of appropriate diabetes care quality; diabetes treatment targets

Results

Diabetes prevalence was higher in urban areas in England (7.80% [95% CI 7.30%; 8.31%]) relative to rural areas (7.24% [95% CI 6.32%; 8.16%]). For practices in urban areas, relatively fewer cases of diabetes were recorded in administrative medical records (69.55% vs. 71.86%), and a smaller percentage of those registered received the appropriate care (45.85% vs. 49.32%). However, practices in urban areas achieved a higher percentage of those receiving appropriate care then achieving treatment targets (77.88% vs. 71.01%).

Analyses adjusting for practice population needs and supply factors showed that the performance of practices in urban areas in providing appropriate care quality was not significantly different from practices in rural areas.

Conclusions

The 'rule of halves' is a heuristic and is useful in drawing attention to gaps that occur across the different aspects of the care pathway, but it is not an accurate description of the actual

For peer review only - http://bmjopen.bmj.com/site/about/guidelines.xhtml

pattern in England. Performance is considerably better than halving in terms of diagnoses in care records and achievement of treatment targets. Overall performance across the levels of the rule of halves is lower for urban areas, but differences are explained by population characteristics and supply factors.

Strengths and limitations of this study

- We estimated the 'rule of halves' for the diabetes care pathway in England for 2016-17 by combining data sources, the first recent demonstration in the UK
- We estimated differences across the levels of the rule of halves in urban and rural settings in England
- We examined the extent to which differences in performance across the levels were explained by population characteristics and supply factors
- Practices returning data to the NDA may be a selected sample and this could vary between the urban and rural CCGs practices
- Patients can opt-out from the NDA and may represent a selected sample

For peer review only - http://bmjopen.bmj.com/site/about/guidelines.xhtml

INTRODUCTION

There are 55.9 million people in the United Kingdom (UK) residing in urban areas, 83.6% of the population (1). Since 1960, the urban population of the UK has grown by 14.8 million and the rural population has decreased by around 370,000 (1). These national trends reflect wider global changes (2). Demographic shifts towards urban areas have implications that include changes in residents' occupational profiles and health behaviours, and for non-communicable chronic illnesses such as diabetes (3,4).

Previous evidence has shown that more than 70% of people with diabetes live in urban areas (5). Conventional risk-factors such as body mass index (BMI), age, diet, family diabetes history and education do not fully explain differences in the diabetes risk of different economic groups of urban-residing individuals (6). The Cities Changing Diabetes (CCD) programme was established in 2014 to generate further understanding of the burden of diabetes and its social and cultural determinants across a range of cities globally (4).

One component of CCD is to describe the pattern of diabetes in terms of: prevalence; diagnosis; receipt of quality care; achievement of treatment targets; and diabetes health outcomes. The 'rule of halves' (ROH) derives from the observation that numbers have been observed to approximately halve at each level from prevalence to outcomes (7), suggesting gaps in appropriate identification, management and treatment of diabetes along the care pathway.

In practice, the ROH represents a broad 'rule of thumb'. Whilst the 'halving' was found to apply for the British NHS in the 1980s for chronic illnesses including diabetes, hypertension and asthma (8), more recent studies suggest that the ROH varies between countries for diabetes. Evidence from low and middle-income countries demonstrates this divergence. For example, estimates from India suggests a rule of 'two-thirds' (i.e. a better performance than halving across levels) (9), whereas evidence from Peru comparing ROH patterns in urban and rural settings suggests considerably worse performance than halving on both care quality and treatment targets (10). Applications of the ROH from high-income settings are equally inconsistent: evidence from Denmark outlined performance far in excess of 'halving' – in particular for diagnosing diabetes and providing treatment (11). Whereas evidence from Australia indicated that the ROH does in fact generally apply for diabetes care and management (12).

For peer review only - http://bmjopen.bmj.com/site/about/guidelines.xhtml

Recent studies have updated historical evidence on the detailed pattern of the ROH for other chronic illnesses such as hypertension and osteoarthritis in England (13–15). However, there are no recent efforts to update historical evidence on the ROH for England despite innovations in diabetes care and prevention over recent decades (16–18). Additionally, despite the urban focus within the CCD programme and its corresponding research aims, the degree to which the ROH varies between urban and rural settings is not well-established for England.

In some instances, studies estimating the ROH have examined how performance across each level varies according to conventional clinical risk factors such age, sex and comorbidity in addition to other population characteristics such as socio-economic factors (e.g. employment status) (10,11). Yet there is limited evidence as to how supply factors (such as available resources in primary care) - which can confound socio-economic influences (19) - impact on performance across levels of the ROH.

In this study, we make three contributions. First, we update estimates on the pattern of the ROH for diabetes in England. Second, we quantify the extent to which the ROH and its subdomains represent an urban phenomenon in England (if at all) by comparing the ROH for urban and rural England. Finally, we examine the extent to which any urban/rural differences in the provision of appropriate care quality and achievement of diabetes care treatment targets can be explained by area-level differences in supply factors in addition to practice population characteristics.

DATA

The ROH requires data on: diabetes prevalence rates and the estimated size of the population; the number of individuals with a recorded diagnosis of diabetes in administrative records; the number of individuals receiving appropriate diabetes care quality; and the number of individuals achieving appropriate diabetes treatment targets.

Diabetes prevalence

We obtained estimated prevalence of diabetes using individual-level data at wave 7 of the UK Household Longitudinal Survey (UKHLS) (covering 2015-2017) (20). The survey started in

For peer review only - http://bmjopen.bmj.com/site/about/guidelines.xhtml

2009, initially comprising 40,000 households across the UK. The survey captures detailed information on mental and physical health in addition to a range of other topics including on urbanity/rurality of respondents' households. It contains large sample of individuals and its sampling methodology allows for examination of a nationally representative group of individuals. We used data for England only to derive an estimate of the mean annual prevalence of diabetes in England for the period 2015-17.

Total population estimates and rural/urban locations

We used estimates of population size at Clinical Commissioning Group (CCG) level by single year of age and sex for England in mid-2016 from the Office for National Statistics (ONS) (21). CCGs are responsible for the commissioning of health services for a defined regional area in England. We combined CCG population size data with ONS geographic data to capture urban and rural spread (22). Additional detail on these data is provided in the appendix.

Diabetes registrations, care quality and treatment target achievement

We obtained practice-level data for England in 2016-17 from the National Diabetes Audit (NDA) (23). NDA measures the effectiveness of diabetes care against National Institute for Health and Care Excellence (NICE) Clinical Guidelines and Quality Standards in England and Wales (24). These data contain information on the number of individuals: with a diabetes diagnosis recorded in administrative records; receiving appropriate care quality; and achieving appropriate treatment targets.

Care quality and treatment target achievement are captured in the NDA via indicators of eight care processes (which should be provided in line with clinical guidelines), and indicators of three treatment targets (that are appropriate for people with diabetes) (Table 1). There are then two summary indicators of the numbers of patients achieving all eight care processes and three treatment targets respectively. Additional details are provided in the appendix.

For peer review only - http://bmjopen.bmj.com/site/about/guidelines.xhtml

Table 1: Appropriate Care Processes and Treatment Targets recorded in the NDA

**NICE recommended Annual Care Processes**	
Process	Detail
HbA1c	blood test for glucose control
Blood Pressure	measurement for cardiovascular risk
Serum Cholesterol	blood test for cardiovascular risk
Serum Creatinine	blood test for kidney function
Urine Albumin/Creatinine Ratio	urine test for early kidney disease
Foot Risk Surveillance	foot examination for foot ulcer risk
Body Mass Index	measurement for diabetes management
Smoking History	question for cardiovascular risk
**NICE recommended Treatment Targets**	
Target	Rationale
HbA1c < 58 mmol/mol	target HbA1c reduces the risk of all diabetic complications
Blood pressure < 140/80	target blood pressure reduces the risk of cardiovascular complications and reduces the progression of eye disease and kidney disease
Cholesterol < 5mmol/L	target cholesterol reduces the risk of cardiovascular complications

Supply factors and practice population characteristics

Supply factors and practice population characteristics were measured using general practice list and workforce data for mid-2016-17 (25). These data record numbers and details of general practitioners (GPs) in England along with information on their practices, staff, and patients. Specifically, the data record general practices' list size and the age/sex composition of their registered populations. These demographics are important determinants of need. The data also provide information on the number of full-time equivalent (FTE) GPs, nurses and administrative staff. These measures allow for the capture of supply factors critical to meeting the need for primary care arising in registered populations.

METHODS

For peer review only - http://bmjopen.bmj.com/site/about/guidelines.xhtml

Populating the levels of the ROH for urban and rural CCGs

Estimating mean diabetes prevalence for England (2015-2017)

We combined data from wave 7 (2015-17) of the UKHLS on participants' self-reports of health conditions with information from a nurse visit assessment in waves 2 and 3 (2010-12) including measurement of glycated haemoglobin (HbA1c). We used this information to create an indicator of whether a participants had diabetes or not at wave 7 to align with the time period covered by the other data sources in this study.

We then used the UKHLS measure of urbanity/rurality of respondents' area of residence to stratify individuals into two groups (urban or rural). This measure classifies respondents addresses as falling into either an urban or rural area based on ONS Rural and Urban Classification of Output Areas (26). In practice this means that respondents are classified as urban if their address falls within urban settlements with a population of 10,000 or more.

We estimated prevalence of diabetes in these two groups at wave 7 (2015-17) including corresponding confidence intervals, applying appropriate longitudinal sample weights (27).

Assessing diabetes registrations, care quality and treatment target achievement

We aggregated data from the NDA at practice-level to CCG-level. These data provide the number of: diabetes registrations; patients receiving all eight care processes; and patients achieving all three treatment targets. We excluded (N=38) CCGs classed as "urban with significant rural" to allow for comparison between more distinctly urban and rural CCGs.

This provided totals of each for the (N=121) predominantly urban and (N=32) predominantly rural CCGs in England. These are summarised in descriptive tables and figures in the supplementary material,

The ROH at national-level in urban and rural areas

To estimate total population prevalence in urban and rural settings in England, we aggregated mid-2016 population estimates by age and sex to total figures for (N=121) predominantly urban and (N=32) predominantly rural CCGs in England.

For peer review only - http://bmjopen.bmj.com/site/about/guidelines.xhtml

However, we applied an adjustment to these population estimates in order to produce a measure of total population prevalence in mid-2016 that would be comparable with the administrative data on diabetes registrations, care quality and treatment target achievement. Practice participation in the NDA is on average 95.3 per cent, and this varies slightly across individual CCGs. We therefore weighted estimated CCG population size by the corresponding figure for practice participation in the NDA. This allowed for consistent comparison of performance between total prevalence and the other levels of the ROH in predominantly urban and predominantly rural CCGs.

For peer review only

Adjusted regression analyses of variation in care quality provision and treatment target achievement

Assessing variation in appropriate care quality between urban and rural CCGs and the influence of population features and supply factors

We used regression analyses to examine whether the percentage of registered people with diabetes receiving all eight care processes varied between general practices in predominantly urban and predominantly rural CCGs, and to what extent any difference might been explained by variation in urban and rural practices' population characteristics and workforce. We include additional variables iteratively to clearly show the impact of including additional controls on the main estimate of interest (the binary indicator for practice membership of a predominantly urban CCG).

First, we included a binary indicator as to whether a practice was located in a predominantly urban CCG.

We then included the characteristics of practices' diabetic and overall populations (binary indicators for the size of the registered diabetic population [under 250; 500 to 749; 750 and over (with 250 to 500 as the reference)]; % of diabetics in age intervals [% under 40; % 40 to 64; % 65 to 79]; % male; % of diabetics from most deprived 40% neighbourhoods based on Index of Multiple Deprivation (IMD) score; total practice list size (divided by 1,000) and the number of patients by sex in each of the following age groups [under 44; 45 to 64; 65 to 84; 85 and over]).

Finally, we included the number of FTE GPs, nurses and administrative staff per 1,000 patients.

For peer review only - http://bmjopen.bmj.com/site/about/guidelines.xhtml

Assessing variation in treatment target achievement between urban and rural CCGs and the influence of population features and supply factors

We then repeated the above analyses instead using the percentage of registered diabetic patients achieving all three treatment targets as the outcome variable.

First, we included the indicator for predominantly urban CCGs, and then included the percentage of registered diabetic patients receiving all eight care processes to control for this component of the ROH. We then included the same measures of practice population characteristics and workforce supply detailed above.

All regression analyses were performed using weighted ordinary least squares at practice-level. The numbers of registered diabetics at practice-level were used as weights, and models were clustered by general practice. We excluded n=42 observations on N=19 practices from the NDA data for which we had no data on workforce/list characteristics from the regression analyses.

Patient and public involvement

Patients and the public were not involved in this study, but results were fed back via community engagement work undertaken as part of a wider research project which includes qualitative work informed by patient engagement.

For peer review only - http://bmjopen.bmj.com/site/about/guidelines.xhtml

RESULTS

The ROH at national-level

The corresponding aggregation of the CCG levels of the ROH to national-level for urban and rural areas are set out in Table 2 and Figure 1.

Table 2: National levels of the ROH by urban/rural status

	Predominantly Urban CCGs (N=121)			Predominantly Rural CCGs (N=32)		
	Estimated total population* = 32,877,630			Estimated total population* = 8,699,248		
	Total	% of previous level**	% of prevalence	Total	% of previous level**	% of prevalence
Diabetes prevalence	2,566,014	7.80%	100.00%	627,431	7.24%	100.00%
Registrations	1,784,715	69.55%	69.55%	450,850	71.86%	71.86%
Appropriate care quality	818,300	45.85%	31.89%	222,345	49.32%	35.44%
Achieve treatment targets	637,325	77.88%	24.84%	157,880	71.01%	25.16%

Notes: *Total population adjusted for practice participation in NDA; diabetes prevalence estimated using UKHLS data for Wave 7 (2015-17); **previous level refers to row above (as denominator), prevalence refers to as % of estimated total population

Aggregation of the urban and rural picture to national-level outlines the pattern of the ROH in each setting. In predominantly urban areas, 69.55% of total estimated prevalence is registered with a diagnosis in administrative records (1.78 million of 2.56 million) (Table 2). This compares with higher overall rates of registration in rural areas (71.86% [450,850 of 627,431]).

In total, practices in urban areas provided appropriate care for 45.85% of patients with a record of diabetes (813,000 of 1.78 million) (Table 2). Overall, practices in rural areas provided 49.32% of patients with a diabetes record with appropriate care (222,345 of 450,850). The relatively lower rate of providing appropriate care in urban areas compounds

For peer review only - http://bmjopen.bmj.com/site/about/guidelines.xhtml

the lower rate of registration in the prevalent population: 31.89% of estimated prevalence receives appropriate care for urban areas, compared with 35.33% in rural areas (in relative terms, a difference of 9.74%).

[EMBED FIGURE 1 HERE]

Finally, practices in urban areas had patients with a record of diabetes achieve their treatment targets at an overall rate of 77.88% amongst those receiving the appropriate care (637,325 of 818,300). This compares with an equivalent rate of 71.01% in rural areas (157,880 of 222.345). However, this higher overall performance for urban areas amongst those receiving appropriate care reflects both lower rates of registration and provision of appropriate care in urban areas. Amongst estimated prevalence, urban areas have 24.84% achieving these targets (637,325 of 2.56 million); compared with 25.16% in rural areas (157,880 of 627,431).

Adjusted analyses of variation in appropriate care quality between urban and rural CCGs

Predominantly urban CCGs had 3.37% fewer patients receiving all eight care processes relative to predominantly rural CCGs ($p<0.001$) (Table 3). However, this difference fell to 1.908% ($p<0.05$) after adjusting for the effect of the characteristics of the practice overall and the diabetic population (Table 3). Finally, there was no statistically significant difference in the performance of predominantly urban and rural practices after further adjusting for indicators of practice workforce supply.

Practices with fewer than 250 patients registered with a record of diabetes had 3.58% ($p<0.001$) fewer patients achieving all eight care processes compared with practices with between 250 and 499 patients with a diabetes record (Table 3). Practices with larger list sizes had fewer patients receiving all eight care processes: 0.54% for each additional 1,000 patients ($p<0.001$).

For peer review only - http://bmjopen.bmj.com/site/about/guidelines.xhtml

For peer review only

Table 3: Regression estimates - analyses of the % of patients receiving appropriate diabetes care quality in general practices

	I	II	III
**Urban / Rural status (reference = predominantly rural)**			
Predominantly Urban = 1	-3.370***	-1.908*	-1.488
**Practice diabetic population characteristics**			
Registered diabetics (< 250) = 1		-3.340***	-3.576***
Registered diabetics (500-749) = 1		2.217**	2.379**
Registered diabetics (750 +) = 1		2.897*	3.029*
% of diabetics aged under 40		0.209	0.236*
% of diabetics aged 40 - 64		-0.071	-0.069
% of diabetics aged 65 - 79		0.122	0.134
% of diabetics = male		0.047	0.066
% of patients from most deprived 40% neighbourhoods		0.043**	0.043**
% of patients from ethnic minorities		-0.090***	-0.088***
% of patients of unknown ethnicity		-0.063***	-0.061***
List size (1,000s)		-0.065	-0.545***
**Practice labour supply**			
GPs per patient			0.530***
Nurses per patient			0.255
Admin staff per patient			0.023
Constant	49.392***	48.814***	48.731***
R-sq.	0.005	0.042	0.049
n	4,913	4,913	4,913

* p<0.05; ** p<0.01; *** p<0.001; Model I = Urban indicator only; Model II = I + Practice population characteristics; Model III = II + Practice labour supply; Models II and III include indicators for the age structure of practice list (by sex); all models estimated at practice-level using OLS weighted by the number of registered diabetics; all models clustered by general practice

Each additional GP per 1,000 patients was associated with a 0.53% increase in registered diabetic patients achieving all eight care processes (Table 3).

For peer review only - http://bmjopen.bmj.com/site/about/guidelines.xhtml

Analyses of variation in treatment target achievement between urban and rural CCGs

Predominantly urban CCGs had 0.755% more patients recorded as achieving all three treatment targets relative to predominantly rural CCGs (p<0.001) (Table 4).

Table 4: Regression estimates - analyses of the % of (registered) diabetic patients achieving diabetes treatment targets in general practices

	I	II	III	IV
**Urban / rural status (reference = predominantly rural)**				
Predominantly Urban = 1	0.755**	1.044***	1.402***	1.309***
**Appropriate care quality**				
% of patients receiving appropriate care		0.086***	0.087***	0.088***
**Practice diabetic population characteristics**				
Registered diabetics (< 250) = 1			1.638***	1.676***
Registered diabetics (500-749) = 1			-0.593*	-0.614*
Registered diabetics (750 +) = 1			-0.04	-0.015
% of diabetics aged under 40			-0.231***	-0.232***
% of diabetics aged 40 - 64			0.052	0.053
% of diabetics aged 65 - 79			0.260***	0.258***
% of diabetics = male			-0.019	-0.021
% of patients from most deprived 40% neighbourhoods			-0.009	-0.009
% of patients from ethnic minorities			0.030***	0.029***
% of patients of unknown ethnicity			-0.025***	-0.025***
List size (1,000s)			-0.086*	-0.022
**Practice labour supply**				
GPs per patient				-0.031
Nurses per patient				-0.069
Admin staff per patient				-0.011
Constant	35.005***	30.781***	34.512***	7.971
R-sq.	0.002	0.054	0.128	0.129
n	4,913	4,913	4,913	4,913

* p<0.05; ** p<0.01; *** p<0.001; Model I = Urban indicator only; Model II = I + Care quality; Model III = II + Practice population characteristics; Model IV = III + Practice labour supply; Models III + IV include indicators for the age structure of practice list (by sex); all models estimated at practice-level using OLS weighted by the number of registered diabetics; all models clustered by general practice

This difference increased to: 1.044% (p<0.001) after adjusting for the effect of the percentage of patients receiving all eight care processes; 1.402% (p<0.001) after adjusting for the

For peer review only - http://bmjopen.bmj.com/site/about/guidelines.xhtml

characteristics of practices overall and diabetic populations; and 1.309% (p<0.001) after including measure of the practice workforce supply (Table 4).

Practices with smaller diabetic populations (fewer than 250 registered) had 1.67% more patients achieving all three treatment targets compared with practices with 250 to 499 patients with a diabetes record (p<0.001). For each additional 10% of patients with a record of diabetes being aged under 40, practices had 2.32% fewer patients achieving all three treatment targets (p<0.001); and for each additional 10% of registered diabetic patients being aged 65 to 79, practices had 2.58% more fewer patients achieving all three treatment targets (p<0.001) (Table 4).

DISCUSSION

Summary of key findings

For practices in predominantly urban areas in England, the ROH displayed a different pattern compared with rural areas for 2016-17. Diabetes prevalence was higher in urban CCGs in England (7.80%) relative to rural areas (7.24%) despite the population being considerably younger. Relatively fewer cases of diabetes were recorded in administrative medical records (69.55% vs. 71.86%), and a smaller percentage of those registered received the appropriate care (45.85% vs. 49.32%). However, practices in urban areas achieved a slightly higher percentage of those receiving appropriate care then achieving treatment targets (24.84% vs. 25.16%). Adjusted analyses showed that the lower performance of practices in urban CCGs in providing appropriate care quality did not persist after adjusting for both the needs of the populations they served and the practice workforce with which they were tasked with meeting those needs.

Explanation of notable findings

Examination of the aggregate performance indicators on provision of appropriate care by practices in urban and rural areas would superficially indicate the provision of a lower level of care quality by urban practices. Yet the adjusted analyses in this study show that these differences do not persist given the populations served and supply factors. In fact, after adjustment: the performance of urban practices was not statistically different in terms of care quality, and was better in terms of treatment target achievement.

For peer review only - http://bmjopen.bmj.com/site/about/guidelines.xhtml

For peer review only

Differences in the proportion of patients with a record of diabetes receiving the appropriate care could in theory reflect differences in attitudes and health literacy amongst urban and rural populations. Practices in urban settings typically serve younger, more deprived and ethnically diverse populations (Supplementary Table S3). Previous studies have shown that education, age, gender and ethnicity have the strongest associations with health literacy in rural and urban areas; and that urbanity/rurality does not represent a specific determinant of health literacy (28). Adjustment for the population characteristics of practices in urban areas substantially reduced the performance gap but did not fully account for the difference. This gap was no longer significant after further adjusting for supply factors. This may reflect inadequate capture of differences in the needs of diabetic populations served by urban and rural practices in the allocation of resources to practices.

Strengths/Comparison with existing research

We updated estimates on the pattern of the ROH in urban and rural England by combining: nationally representative survey data for 2015-2017; demographic and geographic data for mid-2016; and administrative data on diabetes care and treatment targets with national coverage for 2016-17.

We derived an independent estimate of diabetes prevalence in urban and rural areas for England using large, nationally representative survey data. Survey data are well suited to derive estimated disease prevalence for conditions such as diabetes as they minimise the risk of estimates being based on a potentially self-selecting group of individuals such as those in contact with health services (20).

We compared the pattern of the ROH in urban and rural areas to understand the extent to which it may represent an urban phenomenon in England, and at which levels of the ROH the largest differences in performance exist. We then used adjusted regression analyses to examine the extent to which underlying determinants of need and practice workforce supply might explain differences in the performance of practices in urban and rural areas at particular levels of the ROH.

Limitations

For peer review only - http://bmjopen.bmj.com/site/about/guidelines.xhtml

We adjusted population estimates for NDA practice participation to allow for consistency across data sources, and therefore are estimates at an aggregate level will likely understate the totals for each level of the ROH. Practices returning data to the NDA may be a selected sample and this could vary between the urban and rural CCGs practices. Additionally, patients can opt-out from the NDA and may represent a selected sample biasing the NDA practice-level data.

We used a range of data sources to populate the ROH, and these corresponding time periods across data sources was imperfect – for example we used mid-2016 population estimates to compare to 2016-17 data from the NDA. This reflected best availability, but the findings should be interpreted in this context in light of the minor differences between urban and rural areas.

There are five pillars of the ROH, and we excluded the final pillar (diabetes-related health outcomes) from this study due to poor availability of high quality data.

Implications for research and policy

The 'rule of halves' is a heuristic and is useful in drawing attention to how gaps occur across the different aspects of the care pathway, but it is not an accurate description of the actual pattern in England. Performance is considerably better than halving in terms of diagnoses in care records and achievement of treatment targets.

Whilst the differences between English rural and urban settings in terms of performance are relatively small, this does not mean that the causes are the same. Further qualitative research is needed to further understand why drop-offs occur across the levels of the ROH in urban settings.

This evidence could then be used to help inform development of evidence-based health policy and clinical practice for urban populations; for example, by improving health literacy.

For peer review only - http://bmjopen.bmj.com/site/about/guidelines.xhtml

Author contribution:

PB, WW and JD secured the funding. TM, WW, JD and PB designed the analysis. TM performed the analysis. TM drafted the paper and PB, WW, and JD critically revised it. The corresponding author attests that all listed authors meet the relevant criteria and no other meeting these have been omitted.

REFERENCES

1. Clark D. Urban and rural population of the UK 2019 [Internet]. 2020 [cited 2021 Apr 19]. Available from: https://www.statista.com/statistics/984702/urban-and-rural-population-of-the-uk/

2. International Diabetes Federation. IDF diabetes atlas. Brussels: International Diabetes Federation; 2015.

3. Alirol E, Getaz L, Stoll B, Chappuis F, Loutan L. Urbanisation and infectious diseases in a globalised world. Lancet Infect Dis. 2011 Feb 1;11(2):131–41.

4. Napier AD, Nolan JJ, Bagger M, Hesseldal L, Volkmann A-M. Study protocol for the Cities Changing Diabetes programme: a global mixed-methods approach. BMJ Open. 2017 Nov;7(11):e015240.

5. Allender S, Foster C, Hutchinson L, Arambepola C. Quantification of Urbanization in Relation to Chronic Diseases in Developing Countries: A Systematic Review. J Urban Health. 2008 Nov;85(6):938–51.

6. Dagenais GR, Gerstein HC, Zhang X, McQueen M, Lear S, Lopez-Jaramillo P, et al. Variations in Diabetes Prevalence in Low-, Middle-, and High-Income Countries: Results From the Prospective Urban and Rural Epidemiological Study. Diabetes Care. 2016 May 1;39(5):780–7.

7. Mufunda J, Ghebrat Y, Usman A, Mebrahtu G, Gebreslassie A. Underestimation of prevalence of raised blood sugar from history compared to biochemical estimation:

For peer review only - http://bmjopen.bmj.com/site/about/guidelines.xhtml

support for the WHO rule of halves in a population based survey in Eritrea of 2009. SpringerPlus. 2015 Nov 24;4(1):723.

8. Hart JT. Rule of halves: implications of increasing diagnosis and reducing dropout for future workload and prescribing costs in primary care. Br J Gen Pract. 1992 Mar 1;42(356):116–9.

9. Kalra S, Saboo B, Sahay R, Khandelwal D, Talwar V, Unnikrishnan AG. The rule of two-thirds in diabetes epidemiology. Indian J Endocrinol Metab. 2017;21(1):242–4.

10. Lerner AG, Bernabe-Ortiz A, Gilman RH, Smeeth L, Miranda JJ. The 'rule of halves' does not apply in Peru: Awareness, treatment, and control of hypertension and diabetes in rural, urban and rural-to-urban migrants. Crit Pathw Cardiol. 2013 Jun;12(2):53–8.

11. Holm AL, Andersen GS, Jørgensen ME, Diderichsen F. Is the Rule of Halves framework relevant for diabetes care in Copenhagen today? A register-based cross-sectional study. BMJ Open. 2018 Nov;8(11):e023211.

12. Sainsbury E, Shi Y, Flack J, Colagiuri S. The diagnosis and management of diabetes in Australia: Does the "Rule of Halves" apply? Diabetes Res Clin Pract. 2020 Dec 1;170:108524.

13. Wu AS, Dodhia H, Whitney D, Ashworth M. Is the rule of halves still relevant today? A cross-sectional analysis of hypertension detection, treatment and control in an urban community. J Hypertens. 2019 Dec;37(12):2470–2480.

14. Falaschetti E, Chaudhury M, Mindell J, Poulter N. Continued improvement in hypertension management in England: results from the Health Survey for England 2006. Hypertens Dallas Tex 1979. 2009 Mar;53(3):480–6.

15. Sheikh L, Nicholl BI, Green DJ, Bedson J, Peat G. Osteoarthritis and the Rule of Halves. Osteoarthritis Cartilage. 2014 Apr 1;22(4):535–9.

16. French DP, Hawkes RE, Bower P, Cameron E. Is the NHS Diabetes Prevention Programme Intervention Delivered as Planned? An Observational Study of Fidelity of Intervention Delivery. Ann Behav Med [Internet]. 2021 Feb 13 [cited 2021 Apr 19]; Available from: https://academic.oup.com/abm/advance-article/doi/10.1093/abm/kaaa108/6134531

17. Valabhji J, Barron E, Bradley D, Bakhai C, Fagg J, O'Neill S, et al. Early Outcomes From the English National Health Service Diabetes Prevention Programme. Diabetes Care. 2020 Jan 1;43(1):152–60.

18. Chatzi G, Mason T, Chandola T, Whittaker W, Howarth E, Cotterill S, et al. Sociodemographic disparities in non-diabetic hyperglycaemia and the transition to type 2 diabetes: evidence from the English Longitudinal Study of Ageing. Diabet Med. 2020;37(9):1536–44.

19. Jones A, Hayhurst KP, Whittaker W, Mason T, Sutton M. Development of a resource allocation formula for substance misuse treatment services. J Public Health Oxf Engl. 2018 Sep 1;40(3):e396–404.

For peer review only - http://bmjopen.bmj.com/site/about/guidelines.xhtml

20. University of Essex I. United Kingdom Household Longitudinal Study Understanding Society [Internet]. 2019 [cited 2021 Jun 21]. Available from: https://beta.ukdataservice.ac.uk/datacatalogue/doi/?id=6614#!#12

21. ONS. Clinical commissioning group population estimates (National Statistics) [Internet]. 2020 [cited 2021 Jun 21]. Available from: https://www.ons.gov.uk/peoplepopulationandcommunity/populationandmigration/populationestimates/datasets/clinicalcommissioninggroupmidyearpopulationestimates

22. ONS. Rural Urban Classification (2011) of CCGs including population in England [Internet]. 2016 [cited 2021 Jun 21]. Available from: https://geoportal.statistics.gov.uk/datasets/ons::rural-urban-classification-2011-of-ccgs-including-population-in-england/explore

23. NHS Digital. National Diabetes Audit Report 1 Care Processes and Treatment Targets 2016-17 [Internet]. NHS Digital. 2018 [cited 2021 Jun 21]. Available from: https://digital.nhs.uk/data-and-information/publications/statistical/national-diabetes-audit/national-diabetes-audit-report-1-care-processes-and-treatment-targets-2016-17

24. Cartwright C. National Diabetes Audit. 2017;9.

25. NHS Digital. General and Personal Medical Services, England 2006-2016, as at 30 September, Experimental statistics [Internet]. NHS Digital. 2017 [cited 2021 Jun 21]. Available from: https://digital.nhs.uk/data-and-information/publications/statistical/general-practice-workforce-archive/2006-2016-as-at-30-september-experimental-statistics

26. ESRC. Main survey – Understanding Society [Internet]. 2021 [cited 2021 Jun 21]. Available from: https://www.understandingsociety.ac.uk/documentation/mainstage/dataset-documentation/variable/urban_dv

27. UKHLS. Selecting the correct weight for your analysis [Internet]. 2021 [cited 2021 Aug 10]. Available from: https://www.understandingsociety.ac.uk/documentation/mainstage/user-guides/main-survey-user-guide/selecting-the-correct-weight-for-your-analysis

28. Aljassim N, Ostini R. Health literacy in rural and urban populations: A systematic review. Patient Educ Couns. 2020 Oct 1;103(10):2142–54.

For peer review only

SUPPLEMENTARY MATERIAL

DATA

Data on urbanity

The 2011 rural-urban classification (RUC) of clinical commissioning groups (CCG) in England is based on the 2011 RUC of underlying output areas (small-level geographical units). It classifies CCGs into seven classes of the RUC based on the proportion of its population in underlying output areas residing in rural settings. There is also a broader hierarchical grouping of three classes of the RUC which groups CCGs into the following: predominantly rural (at least 50% of the population living in rural settings); predominantly urban (less than 25% of the population living in rural settings; and urban with significant rural (between 25% and 50% of the population living in rural settings).

NDA

Data are extracted from both general practice clinical systems and specialist units in secondary care hospitals (25). The audit is voluntary for general practices and in 2016-17 the participation rate was 95.3 per cent of all practices in England and Wales. For specialist units in secondary care, participation in the audit is compulsory. Whilst the data used in this study are nationally published practice-level data, they are aggregated from individual-level identifiable data from which patients can opt-out.

For peer review only - http://bmjopen.bmj.com/site/about/guidelines.xhtml

METHODS

Estimating mean rates of annual prevalence from the UKHLS

In each wave of the UKHLS, participants are asked various questions about their general health – including questions about whether they have any of a specific set of health conditions including diabetes. The participant then gives information on which conditions apply to them (if any) from the specified list. In addition to these questions about health which are asked in each wave, in waves 2 and 3 (2010-2012) respondents had a nurse health assessment in which a range of biomedical measures were collected including glycated haemoglobin (HbA1c) measurement which is an indicator of diabetes.

We combined these variables to create an indicator of whether an individual had diabetes or not in each wave of the UKHLS, and retained data for wave 7 to align with the time period covered by other data sources in this study.

RESULTS

The levels of the ROH in urban and rural CCGs

Estimated prevalence of diabetes

From the UKHLS we estimated the overall prevalence of diabetes at wave 7 (2015-17) in England as 7.69% (95% CI [7.25%; 81.3%]) (Supplementary Table S1). For those residing in urban settings this was 7.80% (95% CI [7.30%; 8.31%]), compared with 7.24% (95% CI [6.32%; 8.16%]) for those residing in rural settings (Table 2; Supplementary Table S1).

Supplementary Table S1: Estimates of diabetes prevalence for England (2015-17) derived from UKHLS

	N	Mean	95% CI
Overall	33,336	7.69%	[7.25%; 8.13%]
Predominantly Urban	27,374	7.80%	[7.30%; 8.31%]
Predominantly Rural	5,962	7.24%	[6.32%; 8.16%]

For peer review only - http://bmjopen.bmj.com/site/about/guidelines.xhtml

Notes: estimated from UKHLS Wave 7 (2015-2017), longitudinal sample weights applied

Diabetes registrations in the NDA

Predominantly urban CCGs had on average 14,750 people registered as having diabetes ((14,750/21,206); 69.55% of estimated prevalence). This compared to an average of 14,089 in predominantly rural CCGs ((14,089/19,665); 71.64% of estimated prevalence) (Supplementary Table S2).

Provision of appropriate diabetes care quality

On average 6,763 of 14,750 registered people with diabetes in predominantly urban CCGs received all eight appropriate care processes (45.85%) (Supplementary Table S2). In rural settings, this figure was 6,948 of 41,089 registered with diabetes (49.32%).

Achievement of diabetes treatment targets

On average, 5,267 of the 6,763 patients receiving appropriate care achieve diabetes treatment targets in predominantly urban CCGs (77.88%) (Supplementary Table S2). This compares to 4,934 of 6,948 in rural settings (71.01%)

For peer review only - http://bmjopen.bmj.com/site/about/guidelines.xhtml

Supplementary Table S2: Descriptive Statistics for CCGs

		Predominantly Urban CCGs (N=121)		Predominantly Rural CCGs (N=32)	
**Population size and age/sex structure**					
**Variable**		Mean	SD	Mean	SD
Population size		283,640	166,226	285,284	178,929
% Under 40		52.85%	6.47%	43.64%	3.34%
% 40-64		31.25%	2.78%	33.84%	1.18%
% 65-79		11.62%	2.95%	16.56%	2.00%
% 80+		4.28%	1.16%	5.97%	0.74%
% female		50.49%	0.86%	50.72%	0.51%
**Diabetes prevalence, registrations, appropriate care and treatment targets**					
**Variable**		Mean		Mean	
NDA participation of general practices		95.84%		93.83%	
Participation adjusted population size^		271,716		270,914	
Estimated prevalence	Total (in adj. population)**	21,206 [19,847; 22,566]		19,665 [17,115; 22,098]	
	% of population*	7.80% [7.30%; 8.31%]		7.24% [6.32%; 8.16%]	
Registrations	Total	14,750		14,089	
	% of est. prevalence	69.55%		71.64%	
Appropriate care	Total	6,763		6,948	
	% of est. prevalence	31.89%		35.33%	
	% of registrations	45.85%		49.32%	
Treatment targets	Total	5,267		4,934	
	% of est. prevalence	24.84%		25.09%	
	% of registrations	35.71%		35.02%	
	% of appropriate care	77.88%		71.01%	

Notes: *prevalence data estimated from UKHLS Wave 7 (2015-2017), 95% CI in parentheses; ** Total derived from prevalence rates from the UKHLS and population and rurality data from ONS; registrations, quality and treatment from NDA

For peer review only - http://bmjopen.bmj.com/site/about/guidelines.xhtml

Practice population characteristics and workforce supply

Supplementary Table S3 summarises the data on practice population characteristics and workforce supply for predominantly urban and rural CCGs.

Practices in predominantly urban CCGs have 46.02% of those with a diabetes record in receipt of all eight care processes, compared with 49.39% for practices in predominantly rural CCGs (Supplementary Table S3). Practices in predominantly urban CCGs have 46.02% of those with a diabetes record in receipt of all eight care processes, compared with 49.39% for practices in predominantly rural CCGs. Practices in rural CCGs serve older diabetic patients – 45.64% are 65 and older compared with 36.53% in urban settings (Supplementary Table S3).

Practices in predominantly urban CCGs have more of their diabetic patients residing in the most deprived 40% of neighbourhoods (54.2% compared with 31.09% in rural CCGs). Practices in predominantly rural CCGs have a much higher share of diabetic patients (94.99%) with known ethnicity recorded as white compared with those in predominantly urban CCGs (70.54%) (Supplementary Table S3).

Finally, practices in predominantly rural CCGs have more FTE GPs, nurses and administrative staff per 1,000 patients compared with urban CCGs. This is most pronounced in terms of nurses (0.39 per 1,000 for urban vs. 0.51 per 1,000 for rural), 33.15% more – but is still notably bigger in terms of GPs (9.7% more).

For peer review only - http://bmjopen.bmj.com/site/about/guidelines.xhtml

Supplementary Table S3: Care quality, treatment target achievement and practice-level indicators of need and supply in urban and rural areas in England 2016-17

	Predominantly Urban (CCGs=121)	Predominantly Rural (CCGs=32)
Number of General Practices	4,011	902
**Mean Care Quality and Treatment Target Achievement**		
% receiving all care processes	46.02%	49.39%
% achieving all three treatment targets	35.76%	35.01%
**Mean characteristics of registered diabetes and wider practice population**		
Total patients (list size)	7,603	8,931
Total registered diabetics	576	674
Practice prevalence of diabetes (%)	7.57%	7.55%
% registered diabetics male	55.50%	56.71%
% registered diabetics under 40	7.61%	5.94%
% registered diabetics aged 40-64	45.11%	37.72%
% registered diabetics aged 65-79	34.43%	40.17%
% registered diabetics aged 80+	12.10%	15.47%
% registered diabetics from most deprived 40% of neighbourhoods	54.20%	31.09%
% of diabetics with ethnicity unknown	13.01%	23.05%
% of diabetics with known ethnicity = white	70.54%	94.99%
% of diabetics with known ethnicity = minority	29.46%	5.01%
**Mean practice measures of labour supply**		
GPs per 1,000 patients	0.68	0.75
Nurses per 1,000 patients	0.39	0.51
Administrative staff per 1,000 patients	1.63	1.81

Notes: data on diabetes taken from the NDA; data on practice list size and labour supply from GP workforce data from NHS England; data reflect those practices on which complete data exist from both sources

For peer review only - http://bmjopen.bmj.com/site/about/guidelines.xhtml

Figure 1: ROH comparison across levels for urban and rural areas in England (2016-17)

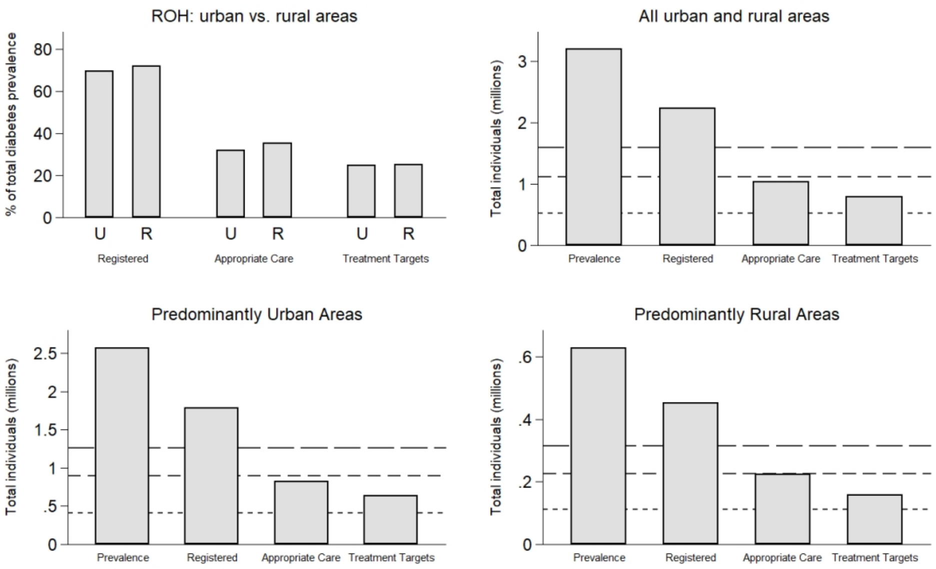

Notes: U=predominantly urban areas, R=predominantly rural areas; long dashed line represents half of actual prevalence, medium dashed line represents half of actual registrations, short dashed line represents half of actual patients receiving appropriate care; prevalence data from UKHLS (2015-17) applied to mid-year ONS population estimates for 2016

494x329mm (72 x 72 DPI)

Reporting checklist for cross sectional study.

Based on the STROBE cross sectional guidelines.

Instructions to authors

Complete this checklist by entering the page numbers from your manuscript where readers will find each of the items listed below.

Your article may not currently address all the items on the checklist. Please modify your text to include the missing information. If you are certain that an item does not apply, please write "n/a" and provide a short explanation.

Upload your completed checklist as an extra file when you submit to a journal.

In your methods section, say that you used the STROBE cross sectionalreporting guidelines, and cite them as:

von Elm E, Altman DG, Egger M, Pocock SJ, Gotzsche PC, Vandenbroucke JP. The Strengthening the Reporting of Observational Studies in Epidemiology (STROBE) Statement: guidelines for reporting observational studies.

		Reporting Item	Page Number
**Title and abstract**			
Title	#1a	Indicate the study’s design with a commonly used term in the title or the abstract	1

For peer review only

Abstract	#1b	Provide in the abstract an informative and balanced summary of what was done and what was found	1, 2
**Introduction**			
Background / rationale	#2	Explain the scientific background and rationale for the investigation being reported	3, 4
Objectives	#3	State specific objectives, including any prespecified hypotheses	4
**Methods**			
Study design	#4	Present key elements of study design early in the paper	7, 8, 9
Setting	#5	Describe the setting, locations, and relevant dates, including periods of recruitment, exposure, follow-up, and data collection	5, 6
Eligibility criteria	#6a	Give the eligibility criteria, and the sources and methods of selection of participants.	n/a
	#7	Clearly define all outcomes, exposures, predictors, potential confounders, and effect modifiers. Give diagnostic criteria, if applicable	7, 8, 9
Data sources / measurement	#8	For each variable of interest give sources of data and details of methods of assessment (measurement). Describe comparability of assessment methods if there is more than one group. Give information separately for for exposed and unexposed groups if applicable.	4, 5, 6

For peer review only

Bias	#9	Describe any efforts to address potential sources of bias	7, 8, 9, 16
Study size	#10	Explain how the study size was arrived at	7, 8, 9
Quantitative variables	#11	Explain how quantitative variables were handled in the analyses. If applicable, describe which groupings were chosen, and why	7, 8, 9
Statistical methods	#12a	Describe all statistical methods, including those used to control for confounding	7, 8, 9
Statistical methods	#12b	Describe any methods used to examine subgroups and interactions	7, 8, 9
Statistical methods	#12c	Explain how missing data were addressed	n/a
Statistical methods	#12d	If applicable, describe analytical methods taking account of sampling strategy	n/a
Statistical methods	#12e	Describe any sensitivity analyses	n/a

Results

Participants	#13a	Report numbers of individuals at each stage of study—eg numbers potentially eligible, examined for eligibility, confirmed eligible, included in the study, completing follow-up, and analysed. Give information separately for for exposed and unexposed groups if applicable.	22 - 26

For peer review only

Participants	#13b	Give reasons for non-participation at each stage	n/a
Participants	#13c	Consider use of a flow diagram	n/a
Descriptive data	#14a	Give characteristics of study participants (eg demographic, clinical, social) and information on exposures and potential confounders. Give information separately for exposed and unexposed groups if applicable.	22 - 26
Descriptive data	#14b	Indicate number of participants with missing data for each variable of interest	n/a
Outcome data	#15	Report numbers of outcome events or summary measures. Give information separately for exposed and unexposed groups if applicable.	n/a
Main results	#16a	Give unadjusted estimates and, if applicable, confounder-adjusted estimates and their precision (eg, 95% confidence interval). Make clear which confounders were adjusted for and why they were included	13, 14, 22, 26
Main results	#16b	Report category boundaries when continuous variables were categorized	13, 14, 24, 26
Main results	#16c	If relevant, consider translating estimates of relative risk into absolute risk for a meaningful time period	n/a
Other analyses	#17	Report other analyses done—e.g., analyses of subgroups and interactions, and sensitivity analyses	22-26

Discussion

For peer review only

Key results	#18	Summarise key results with reference to study objectives	15
Limitations	#19	Discuss limitations of the study, taking into account sources of potential bias or imprecision. Discuss both direction and magnitude of any potential bias.	16, 17
Interpretation	#20	Give a cautious overall interpretation considering objectives, limitations, multiplicity of analyses, results from similar studies, and other relevant evidence.	15, 16
Generalisability	#21	Discuss the generalisability (external validity) of the study results	16, 17
**Other Information**			
Funding	#22	Give the source of funding and the role of the funders for the present study and, if applicable, for the original study on which the present article is based	1, 2

None The STROBE checklist is distributed under the terms of the Creative Commons Attribution License CC-BY. This checklist can be completed online using https://www.goodreports.org/, a tool made by the EQUATOR Network in collaboration with Penelope.ai

For peer review only

BMJ Open

Variation in appropriate diabetes care and treatment targets in urban and rural areas in England: an observational study of the 'rule of halves'

Journal:	*BMJ Open*
Manuscript ID	bmjopen-2021-057244.R1
Article Type:	Original research
Date Submitted by the Author:	25-Nov-2021
Complete List of Authors:	Mason, Thomas; The University of Manchester Faculty of Biology Medicine and Health<br>Whittaker, William; The University of Manchester Faculty of Biology Medicine and Health, Faculty of Biology, Medicine and Health<br>Dumville, Jo C.; The University of Manchester Faculty of Biology Medicine and Health<br>Bower, Peter; The University of Manchester Faculty of Biology Medicine and Health
**Primary Subject Heading**:	Diabetes and endocrinology
Secondary Subject Heading:	Health economics, Epidemiology
Keywords:	Epidemiology < TROPICAL MEDICINE, General diabetes < DIABETES & ENDOCRINOLOGY, Organisation of health services < HEALTH SERVICES ADMINISTRATION & MANAGEMENT, Quality in health care < HEALTH SERVICES ADMINISTRATION & MANAGEMENT

SCHOLARONE™
Manuscripts

I, the Submitting Author has the right to grant and does grant on behalf of all authors of the Work (as defined in the below author licence), an exclusive licence and/or a non-exclusive licence for contributions from authors who are: i) UK Crown employees; ii) where BMJ has agreed a CC-BY licence shall apply, and/or iii) in accordance with the terms applicable for US Federal Government officers or employees acting as part of their official duties; on a worldwide, perpetual, irrevocable, royalty-free basis to BMJ Publishing Group Ltd ("BMJ") its licensees and where the relevant Journal is co-owned by BMJ to the co-owners of the Journal, to publish the Work in this journal and any other BMJ products and to exploit all rights, as set out in our licence.

The Submitting Author accepts and understands that any supply made under these terms is made by BMJ to the Submitting Author unless you are acting as an employee on behalf of your employer or a postgraduate student of an affiliated institution which is paying any applicable article publishing charge ("APC") for Open Access articles. Where the Submitting Author wishes to make the Work available on an Open Access basis (and intends to pay the relevant APC), the terms of reuse of such Open Access shall be governed by a Creative Commons licence – details of these licences and which Creative Commons licence will apply to this Work are set out in our licence referred to above.

Other than as permitted in any relevant BMJ Author's Self Archiving Policies, I confirm this Work has not been accepted for publication elsewhere, is not being considered for publication elsewhere and does not duplicate material already published. I confirm all authors consent to publication of this Work and authorise the granting of this licence.

For peer review only

Variation in appropriate diabetes care and treatment targets in urban and rural areas in England: an observational study of the 'rule of halves'

Thomas Mason[1], Will Whittaker[1], Jo Dumville[1], Peter Bower[1]

[1]*School of Health Sciences; Faculty of Biology, Medicine and Health, University of Manchester, Oxford Road, Manchester, M13 9PL*

Corresponding Author: Thomas Mason

Author email: thomas.mason@manchester.ac.uk; william.whittaker@manchester.ac.uk; jo.dumville@manchester.ac.uk; peter.bower@manchester.ac.uk

The authors of this study have no competing interest to declare

This study did not require ethical approval as it used retrospective, anonymised observational data and all results are reported at an aggregate level

MeSH keywords: diabetes mellitus; urban health; epidemiology; workforce; small-area analysis

Word count (excluding tables, figures and supplement) = 3,726

Funding statement

This research was supported by the National Institute for Health Research Applied Research Collaboration Greater Manchester. The views expressed in this publication are those of the

For peer review only - http://bmjopen.bmj.com/site/about/guidelines.xhtml

author(s) and not necessarily those of the National Institute for Health Research or the Department of Health and Social Care. This research originates from work undertaken in the Cities Changing Diabetes (CCD) programme for Greater Manchester in partnership with and supported by Health Innovation Manchester and Novo Nordisk. CCD was launched in 2014 by the Steno Diabetes Center Copenhagen, University College London, and Novo Nordisk.

Data sharing statement: the population and practice-level data from this study are publicly available from the Office for National Statistics and NHS Digital, the data used to generate prevalence estimates from UK Household Longitudinal Survey can be applied for via the UK Data Service.

For peer review only

For peer review only - http://bmjopen.bmj.com/site/about/guidelines.xhtml

ABSTRACT

Objectives

To estimate the ‘rule of halves’ for diabetes care for urban and rural areas in England using several data sources covering the period 2015-17; and to examine the extent to which any differences in urban and rural settings are explained by population characteristics and the workforce supply of primary care providers (general practices)

Design

A retrospective observational study

Setting

Populations resident in predominantly urban and rural areas in England (2015-17)

Participants

N=33,336 respondents to the UK Household Longitudinal Survey in urban and rural settings in England; N=4,913 general practices in England reporting to the National Diabetes Audit and providing workforce data to NHS Digital

Outcomes

Diabetes prevalence; administrative records of diagnoses of diabetes; provision of (all eight) recommended diabetes care processes; diabetes treatment targets

Results

Diabetes prevalence was higher in urban areas in England (7.80% [95% CI 7.30%; 8.31%]) relative to rural areas (7.24% [95% CI 6.32%; 8.16%]). For practices in urban areas, relatively fewer cases of diabetes were recorded in administrative medical records (69.55% vs. 71.86%), and a smaller percentage of those registered received the appropriate care (45.85% vs. 49.32%). Among estimated prevalent cases of diabetes, urban areas have a 24.84% achieving these targets compared with 25.16% in rural areas.

However, adjusted analyses showed that the performance of practices in urban areas in providing appropriate care quality was not significantly different from practices in rural areas.

For peer review only - http://bmjopen.bmj.com/site/about/guidelines.xhtml

Conclusions

The ‘rule of halves’ is not an accurate description of the actual pattern across the diabetes care pathway in England. More than half of the estimated urban and rural diabetes population are registered with clinical practices and have access to treatment. However, less than half of those registered for treatment have achieved treatment targets.

Appropriate care quality was associated with a greater proportion of diabetic patients achieving treatment targets

For peer review only

Strengths and limitations of this study

- We examined the extent to which differences in performance across the components of the diabetes care pathway were explained by population characteristics and supply factors using adjusted regression analyses
- We estimated urban and rural prevalence of diabetes using nationally representative survey data
- Practices returning data to the National Diabetes Audit (NDA) may be a selected sample and this could vary between the urban and rural practices
- Patients can opt-out from the NDA and may represent a selected sample

For peer review only - http://bmjopen.bmj.com/site/about/guidelines.xhtml

INTRODUCTION

There are 55.9 million people in the United Kingdom (UK) residing in urban areas, 83.6% of the population (1). Since 1960, the urban population of the UK has grown by 14.8 million and the rural population has decreased by around 370,000 (1). These national trends reflect wider global changes (2). Demographic shifts towards urban areas have implications that include changes in residents' occupational profiles and health behaviours, and for non-communicable chronic illnesses such as diabetes (3,4).

Previous evidence has shown that more than 70% of people with diabetes live in urban areas (5). Conventional risk-factors such as body mass index (BMI), age, diet, family diabetes history and education do not fully explain differences in the diabetes risk of different economic groups of urban-residing individuals (6). The Cities Changing Diabetes (CCD) programme was established in 2014 to generate further understanding of the burden of diabetes and its social and cultural determinants across a range of cities globally (4).

One component of CCD is to describe the pattern of diabetes in terms of: prevalence; diagnosis; receipt of quality care; achievement of treatment targets; and diabetes health outcomes. The 'rule of halves' (ROH) derives from the observation that numbers have been observed to approximately halve at each level from prevalence to outcomes (7), suggesting gaps in appropriate identification, management and treatment of diabetes along the care pathway.

In practice, the ROH represents a broad 'rule of thumb' (8): "approximately half of most common chronic disorders are undetected, that half of those detected are not treated, and that half of those treated are not controlled". Whilst the 'halving' was found to apply for the British NHS in the 1980s for chronic illnesses including diabetes, hypertension and asthma (8), more recent studies suggest that the ROH varies between countries for diabetes. Evidence from low and middle-income countries demonstrates this divergence. For example, estimates from India suggests a rule of 'two-thirds' (i.e. a better performance than halving across levels) (9), whereas evidence from Peru comparing ROH patterns in urban and rural settings

For peer review only - http://bmjopen.bmj.com/site/about/guidelines.xhtml

suggests considerably worse performance than halving on both care quality and treatment targets (10). Applications of the ROH from high-income settings are equally inconsistent: evidence from Denmark outlined performance far in excess of 'halving' – in particular for diagnosing diabetes and providing treatment (11). Whereas evidence from Australia indicated that the ROH does in fact generally apply for diabetes care and management (12).

Recent studies have updated historical evidence on the detailed pattern of the ROH for other chronic illnesses such as hypertension and osteoarthritis in England (13–15). However, there are no recent efforts to update historical evidence on the ROH for England despite innovations in diabetes care and prevention over recent decades (16–18). Additionally, despite the urban focus within the CCD programme and its corresponding research aims, the degree to which the ROH varies between urban and rural settings is not well-established for England.

In some instances, studies estimating the ROH have examined how performance across each level varies according to conventional clinical risk factors such age, sex and comorbidity in addition to other population characteristics such as socio-economic factors (e.g. employment status) (10,11). Yet there is limited evidence as to how supply factors (such as available resources in primary care) - which can confound socio-economic influences (19) - impact on performance across levels of the ROH.

In this study, we make three contributions. First, we update estimates on the pattern of the ROH for diabetes in England. Second, we quantify the extent to which the ROH and its subdomains represent an urban phenomenon in England (if at all) by comparing the ROH for urban and rural England. Finally, we examine the extent to which any urban/rural differences in the provision of appropriate care quality and achievement of diabetes care treatment targets can be explained by area-level differences in supply factors in addition to practice population characteristics.

DATA

The ROH requires data on: diabetes prevalence rates and the estimated size of the population; the number of individuals with a recorded diagnosis of diabetes in administrative records; the number of individuals receiving appropriate diabetes care quality; and the number of individuals achieving appropriate diabetes treatment targets.

For peer review only - http://bmjopen.bmj.com/site/about/guidelines.xhtml

Diabetes prevalence

We obtained estimated prevalence of diabetes using individual-level data at wave 7 of the UK Household Longitudinal Survey (UKHLS) (covering 2015-2017) (20). The survey started in 2009, initially comprising 40,000 households across the UK. The survey captures detailed information on mental and physical health in addition to a range of other topics including on urbanity/rurality of respondents' households. It contains large sample of individuals and its sampling methodology allows for examination of a nationally representative group of individuals. We used data for England only to derive an estimate of the mean annual prevalence of diabetes in England for the period 2015-17.

Total population estimates and rural/urban locations

We used estimates of population size at Clinical Commissioning Group (CCG) level by single year of age and sex for England in mid-2016 from the Office for National Statistics (ONS) (21). CCGs are responsible for the commissioning of health services for a defined regional area in England. We combined CCG population size data with ONS geographic data to capture urban and rural spread (22). Additional detail on these data is provided in the appendix.

Diabetes registrations, care quality and treatment target achievement

We obtained practice-level data for England in 2016-17 from the National Diabetes Audit (NDA) (23). NDA measures the effectiveness of diabetes care against National Institute for Health and Care Excellence (NICE) Clinical Guidelines and Quality Standards in England and Wales (24). These data contain information on the number of individuals: with a diabetes diagnosis recorded in administrative records; receiving appropriate care quality; and achieving appropriate treatment targets.

Care quality and treatment target achievement are captured in the NDA via indicators of eight care processes (which should be provided in line with clinical guidelines), and indicators of three treatment targets (that are appropriate for people with diabetes) (Table 1). There are then two summary indicators of the numbers of patients achieving all eight care processes and three treatment targets respectively. Additional details are provided in the appendix.

For peer review only - http://bmjopen.bmj.com/site/about/guidelines.xhtml

Table 1: Appropriate Care Processes and Treatment Targets recorded in the NDA

**NICE recommended Annual Care Processes**	
Process	Detail
HbA1c	blood test for glucose control
Blood Pressure	measurement for cardiovascular risk
Serum Cholesterol	blood test for cardiovascular risk
Serum Creatinine	blood test for kidney function
Urine Albumin/Creatinine Ratio	urine test for early kidney disease
Foot Risk Surveillance	foot examination for foot ulcer risk
Body Mass Index	measurement for diabetes management
Smoking History	question for cardiovascular risk
**NICE recommended Treatment Targets**	
Target	Rationale
HbA1c < 58 mmol/mol	target HbA1c reduces the risk of all diabetic complications
Blood pressure < 140/80*	target blood pressure reduces the risk of cardiovascular complications and reduces the progression of eye disease and kidney disease
Cholesterol < 5mmol/L	target cholesterol reduces the risk of cardiovascular complications

*notes: see (23) and (24). NICE recommendations have been revised since 2016-17 to recommend blood pressure targets at 140/90; and guidelines for best practice/care quality is continuously updated (25)

Supply factors and practice population characteristics

Supply factors and practice population characteristics were measured using general practice list and workforce data for mid-2016-17 (26). These data record numbers and details of general practitioners (GPs) in England along with information on their practices, staff, and patients. Specifically, the data record general practices' list size and the age/sex composition of their registered populations. These demographics are important determinants of need. The data also provide information on the number of full-time equivalent (FTE) GPs, nurses and

For peer review only - http://bmjopen.bmj.com/site/about/guidelines.xhtml

administrative staff. These measures allow for the capture of supply factors critical to meeting the need for primary care arising in registered populations.

METHODS

Populating the levels of the ROH for urban and rural CCGs

Estimating mean diabetes prevalence for England (2015-2017)

We combined data from wave 7 (2015-17) of the UKHLS on participants' self-reports of health conditions with information from a nurse visit assessment in waves 2 and 3 (2010-12) including measurement of glycated haemoglobin (HbA1c). We used this information to create an indicator of whether a participants had diabetes or not at wave 7 to align with the time period covered by the other data sources in this study.

We then used the UKHLS measure of urbanity/rurality of respondents' area of residence to stratify individuals into two groups (urban or rural). This measure classifies respondents addresses as falling into either an urban or rural area based on ONS Rural and Urban Classification of Output Areas (27). In practice this means that respondents are classified as urban if their address falls within urban settlements with a population of 10,000 or more.

We estimated prevalence of diabetes in these two groups at wave 7 (2015-17) including corresponding confidence intervals, applying appropriate longitudinal sample weights (28).

Assessing diabetes registrations, care quality and treatment target achievement

We aggregated data from the NDA at practice-level to CCG-level. These data provide the number of: diabetes registrations; patients receiving all eight care processes; and patients achieving all three treatment targets. Data on care quality from the NDA assume that patients without data for a measurement have not met the criteria for achieving an individual indicator (24). We excluded (N=38) CCGs classed as "urban with significant rural" to allow for comparison between more distinctly urban and rural CCGs (however, these CCGs are included in supplementary analyses).

For peer review only - http://bmjopen.bmj.com/site/about/guidelines.xhtml

This provided totals of each for the (N=121) predominantly urban and (N=32) predominantly rural CCGs in England. These are summarised in descriptive tables and figures in the supplementary material.

The ROH at national-level in urban and rural areas

To estimate total population prevalence in urban and rural settings in England, we aggregated mid-2016 population estimates by age and sex to total figures for (N=121) predominantly urban and (N=32) predominantly rural CCGs in England.

However, we applied an adjustment to these population estimates in order to produce a measure of total population prevalence in mid-2016 that would be comparable with the administrative data on diabetes registrations, care quality and treatment target achievement. Practice participation in the NDA is on average 95.3 per cent, and this varies slightly across individual CCGs. We therefore weighted estimated CCG population size by the corresponding figure for practice participation in the NDA. This allowed for consistent comparison of performance between total prevalence and the other levels of the ROH in predominantly urban and predominantly rural CCGs.

Adjusted regression analyses of variation in care quality provision and treatment target achievement

Assessing variation in appropriate care quality between urban and rural CCGs and the influence of population features and supply factors

We used regression analyses to examine whether the percentage of registered people with diabetes receiving all eight care processes varied between general practices in predominantly urban and predominantly rural CCGs, and to what extent any difference might been explained by variation in urban and rural practices' population characteristics and workforce. We include additional variables iteratively to clearly show the impact of including additional controls on the main estimate of interest (the binary indicator for practice membership of a predominantly urban CCG).

First, we included a binary indicator as to whether a practice was located in a predominantly urban CCG.

For peer review only - http://bmjopen.bmj.com/site/about/guidelines.xhtml

We then included the characteristics of practices' diabetic and overall populations (binary indicators for the size of the registered diabetic population [under 250; 500 to 749; 750 and over (with 250 to 500 as the reference)]; % of diabetics in age intervals [% under 40; % 40 to 64; % 65 to 79]; % male; % of diabetics from most deprived 40% neighbourhoods based on Index of Multiple Deprivation (IMD) score; total practice list size (divided by 1,000) and the number of patients by sex in each of the following age groups [under 44; 45 to 64; 65 to 84; 85 and over]).

Finally, we included the number of FTE GPs, nurses and administrative staff per 1,000 patients.

Assessing variation in treatment target achievement between urban and rural CCGs and the influence of population features and supply factors

We then repeated the above analyses instead using the percentage of registered diabetic patients achieving all three treatment targets as the outcome variable.

First, we included the indicator for predominantly urban CCGs, and then included the percentage of registered diabetic patients receiving all eight care processes to control for this component of the ROH. We then included the same measures of practice population characteristics and workforce supply detailed above.

All regression analyses were performed using weighted ordinary least squares at practice-level. The numbers of registered diabetics at practice-level were used as weights, and models were clustered by general practice. We excluded n=42 observations on N=19 practices from the NDA data for which we had no data on workforce/list characteristics from the regression analyses.

Patient and public involvement

Patients and the public were not involved in this study, but results were fed back via community engagement work undertaken as part of a wider research project which includes qualitative work informed by patient engagement. This study uses retrospective, observational data (most of which are at an aggregated level) and did not require ethical approval.

For peer review only - http://bmjopen.bmj.com/site/about/guidelines.xhtml

For peer review only

RESULTS

A range of supplementary findings are presented and explained in the supplementary material (Supplementary Tables S1-S8; Supplementary Figure 1).

The ROH at national-level

The corresponding aggregation of the CCG levels of the ROH to national-level for urban and rural areas are set out in Table 2 and Figure 1.

Table 2: National levels of the ROH by urban/rural status

	Predominantly Urban CCGs (N=121)			Predominantly Rural CCGs (N=32)		
	Estimated total population* = 32,877,630			Estimated total population* = 8,699,248		
	Total	% of previous level**	% of prevalence	Total	% of previous level**	% of prevalence
Diabetes prevalence	2,566,014	7.80%	100.00%	627,431	7.24%	100.00%
Registrations	1,784,715	69.55%	69.55%	450,850	71.86%	71.86%
Appropriate care quality	818,300	45.85%	31.89%	222,345	49.32%	35.44%

For peer review only - http://bmjopen.bmj.com/site/about/guidelines.xhtml

Achieve treatment targets	637,325	77.88%	24.84%	157,880	71.01%	25.16%

Notes: *Total population adjusted for practice participation in NDA; diabetes prevalence estimated using UKHLS data for Wave 7 (2015-17); **previous level refers to row above (as denominator), prevalence refers to as % of estimated total population

Aggregation of the urban and rural picture to national-level outlines the pattern of the ROH in each setting. In predominantly urban areas, 69.55% of total estimated prevalence is registered with a diagnosis in administrative records (1.78 million of 2.56 million) (Table 2). This compares with higher overall rates of registration in rural areas (71.86% [450,850 of 627,431]).

In total, practices in urban areas provided appropriate care for 45.85% of patients with a record of diabetes (813,000 of 1.78 million) (Table 2). Overall, practices in rural areas provided 49.32% of patients with a diabetes record with appropriate care (222,345 of 450,850). The relatively lower rate of providing appropriate care in urban areas compounds the lower rate of registration in the prevalent population: 31.89% of estimated prevalence receives appropriate care for urban areas, compared with 35.33% in rural areas (in relative terms, a difference of 9.74%).

[EMBED FIGURE 1 HERE]

Finally, practices in urban areas had patients with a record of diabetes achieve their treatment targets at an overall rate of 77.88% amongst those receiving the appropriate care (637,325 of 818,300). This compares with an equivalent rate of 71.01% in rural areas (157,880 of 222.345). However, this higher overall performance for urban areas amongst those receiving appropriate care reflects both lower rates of registration and provision of appropriate care in urban areas. Amongst estimated prevalence, urban areas have 24.84% achieving these targets (637,325 of 2.56 million); compared with 25.16% in rural areas (157,880 of 627,431).

Adjusted analyses of variation in appropriate care quality between urban and rural CCGs

Predominantly urban CCGs had 3.37% fewer patients receiving all eight care processes relative to predominantly rural CCGs (p<0.001) (Table 3). However, this difference fell to

For peer review only - http://bmjopen.bmj.com/site/about/guidelines.xhtml

1.908% (p<0.05) after adjusting for the effect of the characteristics of the practice overall and the diabetic population (Table 3). Finally, there was no statistically significant difference in the performance of predominantly urban and rural practices after further adjusting for indicators of practice workforce supply.

Practices with fewer than 250 patients registered with a record of diabetes had 3.58% (p<0.001) fewer patients achieving all eight care processes compared with practices with between 250 and 499 patients with a diabetes record (Table 3). Practices with larger list sizes had fewer patients receiving all eight care processes: 0.54% for each additional 1,000 patients (p<0.001).

For peer review

Table 3: Regression estimates - analyses of the % of patients receiving appropriate diabetes care quality in general practices

	I	II	III
**Urban / Rural status**			
Predominantly Rural = 1	*reference group*		
Predominantly Urban = 1	-3.370***	-1.908*	-1.488
**Practice diabetic population characteristics**			
Registered diabetics (< 250) = 1		-3.340***	-3.576***
Registered diabetics (250-499) = 1		*reference group*	
Registered diabetics (500-749) = 1		2.217**	2.379**
Registered diabetics (750 +) = 1		2.897*	3.029*
% of diabetics aged under 40		0.209	0.236*
% of diabetics aged 40 - 64		-0.071	-0.069
% of diabetics aged 65 - 79		0.122	0.134
% of diabetics = male		0.047	0.066
% patients from most deprived 40% neighbourhoods		0.043**	0.043**

	I	II	III
% of patients from ethnic minorities		-0.090***	-0.088***
% of patients of unknown ethnicity		-0.063***	-0.061***
List size (1,000s)		-0.065	-0.545***
**Practice labour supply**			
GPs per patient			0.530***
Nurses per patient			0.255
Admin staff per patient			0.023
Constant	49.392***	48.814***	48.731***
R-sq.	0.005	0.042	0.049
n	4,913	4,913	4,913

* p<0.05; ** p<0.01; *** p<0.001; Model I = Urban indicator only; Model II = I + Practice population characteristics; Model III = II + Practice labour supply; Models II and III include indicators for the age structure of practice list (by sex); all models estimated at practice-level using OLS weighted by the number of registered diabetics; all models clustered by general practice

For peer review

Each additional GP per 1,000 patients was associated with a 0.53% increase in registered diabetic patients achieving all eight care processes (Table 3).

Analyses of variation in treatment target achievement between urban and rural CCGs

Predominantly urban CCGs had 0.755% more patients recorded as achieving all three treatment targets relative to predominantly rural CCGs (p<0.001) (Table 4).

Table 4: Regression estimates - analyses of the % of (registered) diabetic patients achieving diabetes treatment targets in general practices

	I	II	III	IV
**Urban / rural status (reference = predominantly rural)**				
Predominantly Rural = 1	*reference group*			
Predominantly Urban = 1	0.755**	1.044***	1.402***	1.309***
**Appropriate care quality**				
% of patients receiving appropriate care		0.086***	0.087***	0.088***
**Practice diabetic population characteristics**				
Registered diabetics (< 250) = 1			1.638***	1.676***
Registered diabetics (250-499) = 1			*reference group*	
Registered diabetics (500-749) = 1			-0.593*	-0.614*
Registered diabetics (750 +) = 1			-0.04	-0.015
% of diabetics aged under 40			-0.231***	-0.232***
% of diabetics aged 40 - 64			0.052	0.053
% of diabetics aged 65 - 79			0.260***	0.258***

% of diabetics = male			-0.019	-0.021
% of patients from most deprived 40% neighbourhoods			-0.009	-0.009
% of patients from ethnic minorities			0.030***	0.029***
% of patients of unknown ethnicity			-0.025***	-0.025***
List size (1,000s)			-0.086*	-0.022
**Practice labour supply**				
GPs per patient				-0.031
Nurses per patient				-0.069
Admin staff per patient				-0.011
Constant	35.005***	30.781***	34.512***	7.971
R-sq.	0.002	0.054	0.128	0.129
n	4,913	4,913	4,913	4,913

* p<0.05; ** p<0.01; *** p<0.001; Model I = Urban indicator only; Model II = I + Care quality; Model III = II + Practice population characteristics; Model IV = III + Practice labour supply; Models III + IV include indicators for the age structure of practice list (by sex); all models estimated at practice-level using OLS weighted by the number of registered diabetics; all models clustered by general practice

This difference increased to: 1.044% (p<0.001) after adjusting for the effect of the percentage of patients receiving all eight care processes; 1.402% (p<0.001) after adjusting for the characteristics of practices overall and diabetic populations; and 1.309% (p<0.001) after including measure of the practice workforce supply (Table 4).

Practices with smaller diabetic populations (fewer than 250 registered) had 1.67% more patients achieving all three treatment targets compared with practices with 250 to 499 patients with a diabetes record (p<0.001). For each additional 10% of patients with a record of diabetes being aged under 40, practices had 2.32% fewer patients achieving all three treatment targets (p<0.001); and for each additional 10% of registered diabetic patients being aged 65 to 79, practices had 2.58% more fewer patients achieving all three treatment targets (p<0.001) (Table 4).

DISCUSSION

Summary of key findings

For practices in predominantly urban areas in England, the ROH displayed a different pattern compared with rural areas for 2016-17. Diabetes prevalence was higher in urban CCGs in England (7.80%) relative to rural areas (7.24%) despite the population being considerably younger. Relatively fewer cases of diabetes were recorded in administrative medical records

For peer review only - http://bmjopen.bmj.com/site/about/guidelines.xhtml

(69.55% vs. 71.86%), and a smaller percentage of those registered received the appropriate care (45.85% vs. 49.32%). However, practices in urban areas achieved a slightly higher percentage of those receiving appropriate care then achieving treatment targets (24.84% vs. 25.16%). Adjusted analyses showed that the lower performance of practices in urban CCGs in providing appropriate care quality did not persist after adjusting for both the needs of the populations they served and the practice workforce with which they were tasked with meeting those needs.

Explanation of notable findings

Examination of the aggregate performance indicators on provision of appropriate care by practices in urban and rural areas would superficially indicate the provision of a lower level of care quality by urban practices. Yet the adjusted analyses in this study show that these differences do not persist given the populations served and supply factors. In fact, after adjustment: the performance of urban practices was not statistically different in terms of care quality, and was better in terms of treatment target achievement.

Differences in the proportion of patients with a record of diabetes receiving the appropriate care could in theory reflect differences in attitudes and health literacy amongst urban and rural populations. Practices in urban settings typically serve younger, more deprived and ethnically diverse populations (Supplementary Table S3). Previous studies have shown that education, age, gender and ethnicity have the strongest associations with health literacy in rural and urban areas; and that urbanity/rurality does not represent a specific determinant of health literacy (29). Adjustment for the population characteristics of practices in urban areas substantially reduced the performance gap but did not fully account for the difference. This gap was no longer significant after further adjusting for supply factors. This may reflect inadequate capture of differences in the needs of diabetic populations served by urban and rural practices in the allocation of resources to practices.

Adjusted analyses confirmed that practices serving rural populations tend to have lower rates of treatment target achievement, even after adjusting for population and workforce characteristics. The data did not allow for exhaustive examination of the possible underlying causes of this. However, there are possible explanations which may warrant further investigation in future research. Patients registered with rurally located practices typically have further to travel to access primary care, and those located furthest away exhibit a lower

For peer review only - http://bmjopen.bmj.com/site/about/guidelines.xhtml

propensity to use services (30–32). Maintenance of appropriately controlled HbA1c levels in patients with diabetes typically requires prescription of metformin, the standard first line of therapy (25). However, in patients who are inappropriately controlled by metformin, intensification of therapy is required (with additional prescription drugs). Future studies might investigate whether rurally residing diabetes patients exhibit a lower propensity to seek out primary care when they are becoming more poorly controlled.

Strengths/Comparison with existing research

We updated estimates on the pattern of the ROH in urban and rural England by combining: nationally representative survey data for 2015-2017; demographic and geographic data for mid-2016; and administrative data on diabetes care and treatment targets with national coverage for 2016-17.

We derived an independent estimate of diabetes prevalence in urban and rural areas for England using large, nationally representative survey data. Survey data are well suited to derive estimated disease prevalence for conditions such as diabetes as they minimise the risk of estimates being based on a potentially self-selecting group of individuals such as those in contact with health services (18).

We compared the pattern of the ROH in urban and rural areas to understand the extent to which it may represent an urban phenomenon in England, and at which levels of the ROH the largest differences in performance exist. We then used adjusted regression analyses to examine the extent to which underlying determinants of need and practice workforce supply might explain differences in the performance of practices in urban and rural areas at particular levels of the ROH.

Limitations

We adjusted population estimates for NDA practice participation to allow for consistency across data sources, and therefore are estimates at an aggregate level will likely understate the totals for each level of the ROH. Practices returning data to the NDA may be a selected sample and this could vary between the urban and rural CCGs practices. Additionally, patients can opt-out from the NDA and may represent a selected sample biasing the NDA practice-level data.

For peer review only - http://bmjopen.bmj.com/site/about/guidelines.xhtml

We used a range of data sources to populate the ROH, and these corresponding time periods across data sources was imperfect – for example we used mid-2016 population estimates to compare to 2016-17 data from the NDA. This reflected best availability, but the findings should be interpreted in this context in light of the minor differences between urban and rural areas. Data on diabetes registrations, care processes and treatment targets were for the financial year 2016-17. However, the guidelines around best practice care are updated continuously and the findings of this study should be interpreted on this basis.

There are five pillars of the ROH, and we excluded the final pillar (diabetes-related health outcomes) from this study due to poor availability of high quality data.

Implications for research and policy

The 'rule of halves' is a heuristic and is useful in drawing attention to how gaps occur across the different aspects of the care pathway, but it is not an accurate description of the actual pattern in England. More than half of the estimated urban and rural diabetes population are registered with clinical practices and have access to treatment. However, less than half of those registered for treatment have achieved treatment targets. The aggregate differences between practices in urban and rural settings are less pronounced after reflecting the differences in the populations served and the workforce used to provide care.

Whilst the differences between English rural and urban settings in terms of performance are relatively small, this does not mean that the causes are the same. Further qualitative research is needed to further understand why drop-offs occur across the levels of the ROH in urban settings. Future studies could also explore whether diabetes patients' health literacy does in fact explain some of the variation between patients residing in urban and rural settings.

This evidence could then be used to help inform development of evidence-based health policy and clinical practice for urban populations; for example, by improving health literacy.

For peer review only - http://bmjopen.bmj.com/site/about/guidelines.xhtml

Author contribution:

PB, WW and JD secured the funding. TM, WW, JD and PB designed the analysis. TM performed the analysis. TM drafted the paper and PB, WW, and JD critically revised it. The corresponding author attests that all listed authors meet the relevant criteria and no other meeting these have been omitted.

Figure Legends

Figure 1 Legend: ROH comparison across levels for urban and rural areas in England (2016-17)

Supplementary Figure S1 Legend: ROH for 'Urban with Significant Rural' Areas

For peer review only

REFERENCES

1. Clark D. Urban and rural population of the UK 2019 [Internet]. 2020 [cited 2021 Apr 19]. Available from: https://www.statista.com/statistics/984702/urban-and-rural-population-of-the-uk/

2. International Diabetes Federation. IDF diabetes atlas. Brussels: International Diabetes Federation; 2015.

3. Alirol E, Getaz L, Stoll B, Chappuis F, Loutan L. Urbanisation and infectious diseases in a globalised world. Lancet Infect Dis. 2011 Feb 1;11(2):131–41.

4. Napier AD, Nolan JJ, Bagger M, Hesseldal L, Volkmann A-M. Study protocol for the Cities Changing Diabetes programme: a global mixed-methods approach. BMJ Open. 2017 Nov;7(11):e015240.

For peer review only - http://bmjopen.bmj.com/site/about/guidelines.xhtml

5. Allender S, Foster C, Hutchinson L, Arambepola C. Quantification of Urbanization in Relation to Chronic Diseases in Developing Countries: A Systematic Review. J Urban Health. 2008 Nov;85(6):938–51.

6. Dagenais GR, Gerstein HC, Zhang X, McQueen M, Lear S, Lopez-Jaramillo P, et al. Variations in Diabetes Prevalence in Low-, Middle-, and High-Income Countries: Results From the Prospective Urban and Rural Epidemiological Study. Diabetes Care. 2016 May 1;39(5):780–7.

7. Mufunda J, Ghebrat Y, Usman A, Mebrahtu G, Gebreslassie A. Underestimation of prevalence of raised blood sugar from history compared to biochemical estimation: support for the WHO rule of halves in a population based survey in Eritrea of 2009. SpringerPlus. 2015 Nov 24;4(1):723.

8. Hart JT. Rule of halves: implications of increasing diagnosis and reducing dropout for future workload and prescribing costs in primary care. Br J Gen Pract. 1992 Mar 1;42(356):116–9.

9. Kalra S, Saboo B, Sahay R, Khandelwal D, Talwar V, Unnikrishnan AG. The rule of two-thirds in diabetes epidemiology. Indian J Endocrinol Metab. 2017;21(1):242–4.

10. Lerner AG, Bernabe-Ortiz A, Gilman RH, Smeeth L, Miranda JJ. The 'rule of halves' does not apply in Peru: Awareness, treatment, and control of hypertension and diabetes in rural, urban and rural-to-urban migrants. Crit Pathw Cardiol. 2013 Jun;12(2):53–8.

11. Holm AL, Andersen GS, Jørgensen ME, Diderichsen F. Is the Rule of Halves framework relevant for diabetes care in Copenhagen today? A register-based cross-sectional study. BMJ Open. 2018 Nov;8(11):e023211.

12. Sainsbury E, Shi Y, Flack J, Colagiuri S. The diagnosis and management of diabetes in Australia: Does the "Rule of Halves" apply? Diabetes Res Clin Pract. 2020 Dec 1;170:108524.

13. Wu AS, Dodhia H, Whitney D, Ashworth M. Is the rule of halves still relevant today? A cross-sectional analysis of hypertension detection, treatment and control in an urban community. J Hypertens. 2019 Dec;37(12):2470–2480.

14. Falaschetti E, Chaudhury M, Mindell J, Poulter N. Continued improvement in hypertension management in England: results from the Health Survey for England 2006. Hypertens Dallas Tex 1979. 2009 Mar;53(3):480–6.

15. Sheikh L, Nicholl BI, Green DJ, Bedson J, Peat G. Osteoarthritis and the Rule of Halves. Osteoarthritis Cartilage. 2014 Apr 1;22(4):535–9.

16. French DP, Hawkes RE, Bower P, Cameron E. Is the NHS Diabetes Prevention Programme Intervention Delivered as Planned? An Observational Study of Fidelity of Intervention Delivery. Ann Behav Med [Internet]. 2021 Feb 13 [cited 2021 Apr 19]; Available from: https://academic.oup.com/abm/advance-article/doi/10.1093/abm/kaaa108/6134531

For peer review only - http://bmjopen.bmj.com/site/about/guidelines.xhtml

17. Valabhji J, Barron E, Bradley D, Bakhai C, Fagg J, O'Neill S, et al. Early Outcomes From the English National Health Service Diabetes Prevention Programme. Diabetes Care. 2020 Jan 1;43(1):152–60.

18. Chatzi G, Mason T, Chandola T, Whittaker W, Howarth E, Cotterill S, et al. Sociodemographic disparities in non-diabetic hyperglycaemia and the transition to type 2 diabetes: evidence from the English Longitudinal Study of Ageing. Diabet Med. 2020;37(9):1536–44.

19. Jones A, Hayhurst KP, Whittaker W, Mason T, Sutton M. Development of a resource allocation formula for substance misuse treatment services. J Public Health Oxf Engl. 2018 Sep 1;40(3):e396–404.

20. University of Essex I. United Kingdom Household Longitudinal Study Understanding Society [Internet]. 2019 [cited 2021 Jun 21]. Available from: https://beta.ukdataservice.ac.uk/datacatalogue/doi/?id=6614#!#12

21. ONS. Clinical commissioning group population estimates (National Statistics) [Internet]. 2020 [cited 2021 Jun 21]. Available from: https://www.ons.gov.uk/peoplepopulationandcommunity/populationandmigration/populationestimates/datasets/clinicalcommissioninggroupmidyearpopulationestimates

22. ONS. Rural Urban Classification (2011) of CCGs including population in England [Internet]. 2016 [cited 2021 Jun 21]. Available from: https://geoportal.statistics.gov.uk/datasets/ons::rural-urban-classification-2011-of-ccgs-including-population-in-england/explore

23. NHS Digital. National Diabetes Audit Report 1 Care Processes and Treatment Targets 2016-17 [Internet]. NHS Digital. 2018 [cited 2021 Jun 21]. Available from: https://digital.nhs.uk/data-and-information/publications/statistical/national-diabetes-audit/national-diabetes-audit-report-1-care-processes-and-treatment-targets-2016-17

24. NHS Digital. National Diabetes Audit Data Quality Statement, 2016-17. 2017;9.

25. NICE. Type 2 diabetes in adults: management [Internet]. 2015 p. 36. Available from: https://www.nice.org.uk/guidance/ng28/resources/type-2-diabetes-in-adults-management-pdf-1837338615493

26. NHS Digital. General and Personal Medical Services, England 2006-2016, as at 30 September, Experimental statistics [Internet]. NHS Digital. 2017 [cited 2021 Jun 21]. Available from: https://digital.nhs.uk/data-and-information/publications/statistical/general-practice-workforce-archive/2006-2016-as-at-30-september-experimental-statistics

27. ESRC. Main survey – Understanding Society [Internet]. 2021 [cited 2021 Jun 21]. Available from: https://www.understandingsociety.ac.uk/documentation/mainstage/dataset-documentation/variable/urban_dv

28. UKHLS. Selecting the correct weight for your analysis [Internet]. 2021 [cited 2021 Aug 10]. Available from:

For peer review only - http://bmjopen.bmj.com/site/about/guidelines.xhtml

https://www.understandingsociety.ac.uk/documentation/mainstage/user-guides/main-survey-user-guide/selecting-the-correct-weight-for-your-analysis

29. Aljassim N, Ostini R. Health literacy in rural and urban populations: A systematic review. Patient Educ Couns. 2020 Oct 1;103(10):2142–54.

30. Turnbull J, Martin D, Lattimer V, Pope C, Culliford D. Does distance matter? Geographical variation in GP out-of-hours service use: an observational study. Br J Gen Pract. 2008 Jul 1;58(552):471–7.

31. Farmer J, Iversen L, Campbell NC, Guest C, Chesson R, Deans G, et al. Rural/urban differences in accounts of patients' initial decisions to consult primary care. Health Place. 2006 Jun;12(2):210–21.

32. Bentham G, Haynes R. Health, personal mobility and the use of health services in rural Norfolk. J Rural Stud. 1985 Jan 1;1(3):231–9.

For peer review only

Figure 1: ROH comparison across levels for urban and rural areas in England (2016-17)

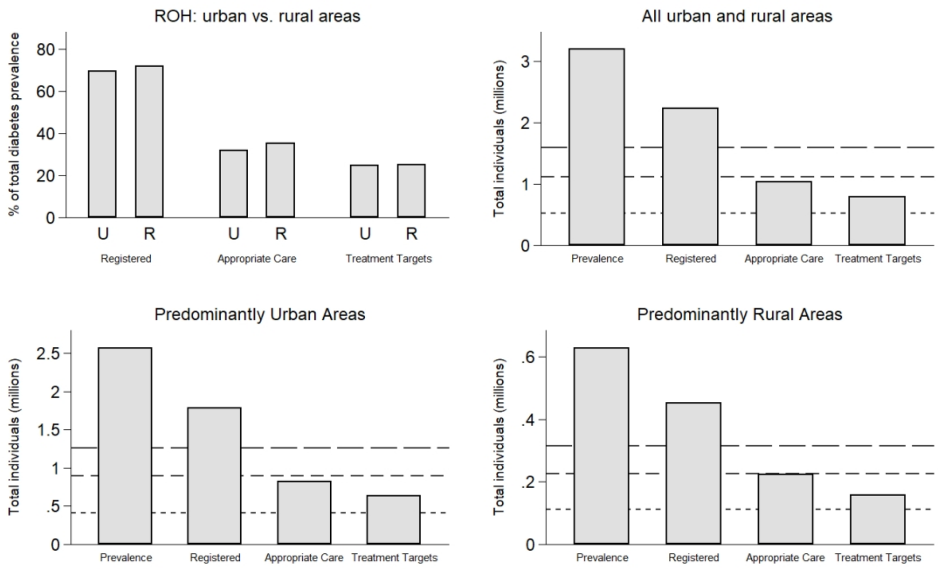

Notes: U=predominantly urban areas, R=predominantly rural areas; long dashed line represents half of actual prevalence, medium dashed line represents half of actual registrations, short dashed line represents half of actual patients receiving appropriate care; prevalence data from UKHLS (2015-17) applied to mid-year ONS population estimates for 2016

494x329mm (72 x 72 DPI)

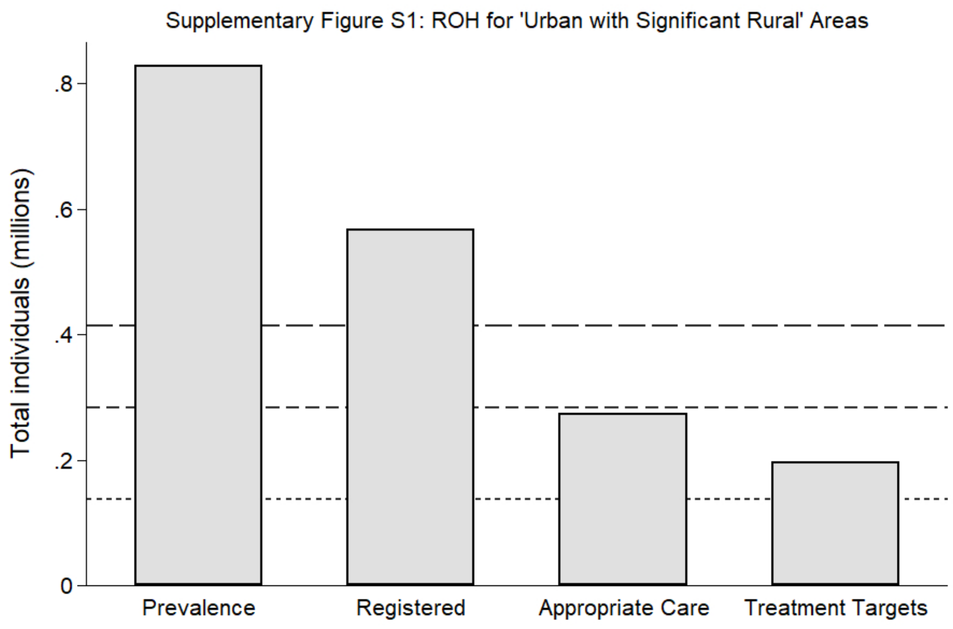

Notes: U=predominantly urban areas, R=predominantly rural areas; long dashed line represents half of actual prevalence, medium dashed line represents half of actual registrations, short dashed line represents half of actual patients receiving appropriate care; prevalence data from UKHLS (2015-17) applied to mid-year ONS population estimates for 2016

327x218mm (72 x 72 DPI)

SUPPLEMENTARY MATERIAL

DATA

Data on urbanity

The 2011 rural-urban classification (RUC) of clinical commissioning groups (CCG) in England is based on the 2011 RUC of underlying output areas (small-level geographical units). It classifies CCGs into seven classes of the RUC based on the proportion of its population in underlying output areas residing in rural settings. There is also a broader hierarchical grouping of three classes of the RUC which groups CCGs into the following: predominantly rural (at least 50% of the population living in rural settings); predominantly urban (less than 25% of the population living in rural settings; and urban with significant rural (between 25% and 50% of the population living in rural settings).

NDA

Data are extracted from both general practice clinical systems and specialist units in secondary care hospitals (24). The audit is voluntary for general practices and in 2016-17 the participation rate was 95.3 per cent of all practices in England and Wales. For specialist units in secondary care, participation in the audit is compulsory. Whilst the data used in this study are nationally published practice-level data, they are aggregated from individual-level identifiable data from which patients can opt-out.

The 2016-17 NDA covers the majority of England (and Wales) with a participation rate of 95.3% (24). Information is collated from GP clinical systems and comparable data are collected from secondary care providers over a six week period. Data on registrations represent counts of patients who have a recorded diagnosis of diabetes in these data returns.

For peer review only - http://bmjopen.bmj.com/site/about/guidelines.xhtml

METHODS

Estimating mean rates of annual prevalence from the UKHLS

In each wave of the UKHLS, participants are asked various questions about their general health – including questions about whether they have any of a specific set of health conditions including diabetes. The participant then gives information on which conditions apply to them (if any) from the specified list. In addition to these questions about health which are asked in each wave, in waves 2 and 3 (2010-2012) respondents had a nurse health assessment in which a range of biomedical measures were collected including glycated haemoglobin (HbA1c) measurement which is an indicator of diabetes.

We combined these variables to create an indicator of whether an individual had diabetes or not in each wave of the UKHLS, and retained data for wave 7 to align with the time period covered by other data sources in this study.

RESULTS

The levels of the ROH in urban and rural CCGs

Estimated prevalence of diabetes

From the UKHLS we estimated the overall prevalence of diabetes at wave 7 (2015-17) in England as 7.69% (95% CI [7.25%; 81.3%]) (Supplementary Table S1). For those residing in urban settings this was 7.80% (95% CI [7.30%; 8.31%]), compared with 7.24% (95% CI [6.32%; 8.16%]) for those residing in rural settings (Table 2; Supplementary Table S1).

Supplementary Table S1: Estimates of diabetes prevalence for England (2015-17) derived from UKHLS

	N	Mean	95% CI
Overall	33,336	7.69%	[7.25%; 8.13%]
Predominantly Urban	27,374	7.80%	[7.30%; 8.31%]
Predominantly Rural	5,962	7.24%	[6.32%; 8.16%]

For peer review only - http://bmjopen.bmj.com/site/about/guidelines.xhtml

Notes: estimated from UKHLS Wave 7 (2015-2017), longitudinal sample weights applied

Diabetes registrations in the NDA

Predominantly urban CCGs had on average 14,750 people registered as having diabetes ((14,750/21,206); 69.55% of estimated prevalence). This compared to an average of 14,089 in predominantly rural CCGs ((14,089/19,665); 71.64% of estimated prevalence) (Supplementary Table S2).

Provision of appropriate diabetes care quality

On average 6,763 of 14,750 registered people with diabetes in predominantly urban CCGs received all eight appropriate care processes (45.85%) (Supplementary Table S2). In rural settings, this figure was 6,948 of 41,089 registered with diabetes (49.32%).

Achievement of diabetes treatment targets

On average, 5,267 of the 6,763 patients receiving appropriate care achieve diabetes treatment targets in predominantly urban CCGs (77.88%) (Supplementary Table S2). This compares to 4,934 of 6,948 in rural settings (71.01%)

For peer review only

Supplementary Table S2: Descriptive Statistics for CCGs

		Predominantly Urban CCGs (N=121)		Predominantly Rural CCGs (N=32)	
**Population size and age/sex structure**					
**Variable**		Mean	SD	Mean	SD
Population size		283,640	166,226	285,284	178,929
% Under 40		52.85%	6.47%	43.64%	3.34%
% 40-64		31.25%	2.78%	33.84%	1.18%
% 65-79		11.62%	2.95%	16.56%	2.00%
% 80+		4.28%	1.16%	5.97%	0.74%
% female		50.49%	0.86%	50.72%	0.51%
**Diabetes prevalence, registrations, appropriate care and treatment targets**					
**Variable**		Mean		Mean	
NDA participation of general practices		95.84%		93.83%	
Participation adjusted population size^		271,716		270,914	
Estimated prevalence	Total (in adj. population)**	21,206 [19,847; 22,566]		19,665 [17,115; 22,098]	
	% of population*	7.80% [7.30%; 8.31%]		7.24% [6.32%; 8.16%]	
Registrations	Total	14,750		14,089	
	% of est. prevalence	69.55%		71.64%	
Appropriate care	Total	6,763		6,948	
	% of est. prevalence	31.89%		35.33%	
	% of registrations	45.85%		49.32%	
Treatment targets	Total	5,267		4,934	
	% of est. prevalence	24.84%		25.09%	
	% of registrations	35.71%		35.02%	
	% of appropriate care	77.88%		71.01%	

Notes: *prevalence data estimated from UKHLS Wave 7 (2015-2017), 95% CI in parentheses; ** Total derived from prevalence rates from the UKHLS and population and rurality data from ONS; registrations, quality and treatment from NDA

For peer review only - http://bmjopen.bmj.com/site/about/guidelines.xhtml

Practice population characteristics and workforce supply

Supplementary Table S3 summarises the data on practice population characteristics and workforce supply for predominantly urban and rural CCGs.

Practices in predominantly urban CCGs have 46.02% of those with a diabetes record in receipt of all eight care processes, compared with 49.39% for practices in predominantly rural CCGs (Supplementary Table S3). Practices in predominantly urban CCGs have 46.02% of those with a diabetes record in receipt of all eight care processes, compared with 49.39% for practices in predominantly rural CCGs. Practices in rural CCGs serve older diabetic patients – 45.64% are 65 and older compared with 36.53% in urban settings (Supplementary Table S3).

Practices in predominantly urban CCGs have more of their diabetic patients residing in the most deprived 40% of neighbourhoods (54.2% compared with 31.09% in rural CCGs). Practices in predominantly rural CCGs have a much higher share of diabetic patients (94.99%) with known ethnicity recorded as white compared with those in predominantly urban CCGs (70.54%) (Supplementary Table S3).

Finally, practices in predominantly rural CCGs have more FTE GPs, nurses and administrative staff per 1,000 patients compared with urban CCGs. This is most pronounced in terms of nurses (0.39 per 1,000 for urban vs. 0.51 per 1,000 for rural), 33.15% more – but is still notably bigger in terms of GPs (9.7% more).

For peer review only - http://bmjopen.bmj.com/site/about/guidelines.xhtml

Supplementary Table S3: Care quality, treatment target achievement and practice-level indicators of need and supply in urban and rural areas in England 2016-17

	Predominantly Urban (CCGs=121)	Predominantly Rural (CCGs=32)
Number of General Practices	4,011	902
**Mean Care Quality and Treatment Target Achievement**		
% receiving all care processes	46.02%	49.39%
% achieving all three treatment targets	35.76%	35.01%
**Mean characteristics of registered diabetes and wider practice population**		
Total patients (list size)	7,603	8,931
Total registered diabetics	576	674
Practice prevalence of diabetes (%)	7.57%	7.55%
% registered diabetics male	55.50%	56.71%
% registered diabetics under 40	7.61%	5.94%
% registered diabetics aged 40-64	45.11%	37.72%
% registered diabetics aged 65-79	34.43%	40.17%
% registered diabetics aged 80+	12.10%	15.47%
% registered diabetics from most deprived 40% of neighbourhoods	54.20%	31.09%
% of diabetics with ethnicity unknown	13.01%	23.05%
% of diabetics with known ethnicity = white	70.54%	94.99%
% of diabetics with known ethnicity = minority	29.46%	5.01%
**Mean practice measures of labour supply**		
GPs per 1,000 patients	0.68	0.75
Nurses per 1,000 patients	0.39	0.51
Administrative staff per 1,000 patients	1.63	1.81

Notes: data on diabetes taken from the NDA; data on practice list size and labour supply from GP workforce data from NHS England; data reflect those practices on which complete data exist from both sources

review only

Extension to include 'urban with significant rural' CCGs

Supplementary Table S4: National levels of the ROH by urban/rural status (including "urban with significant rural" CCGs)

	Predominantly Urban CCGs (N=121)			Predominantly Rural CCGs (N=32)			Urban with Significant Rural CCGs (N=38)		
	Estimated total population* = 32,877,630			Estimated total population* = 8,699,248			Estimated total population* = 10,914,316		
	Total	% of previous level**	% of prevalence	Total	% of previous level**	% of prevalence	Total^	% of previous level**	% of prevalence
Diabetes prevalence	2,566,014	7.80%	100.00%	627,431	7.24%	100.00%	828,615	7.59%	100.00%
Registrations	1,784,715	69.55%	69.55%	450,850	71.86%	71.86%	567,400	68.48%	68.48%
Appropriate Care Quality	818,300	45.85%	31.89%	222,345	49.32%	35.44%	273,205	48.15%	32.97%
Achieve Treatment Targets	637,325	77.88%	24.84%	157,880	71.01%	25.16%	195,750	71.65%	23.62%

Notes: *Total population adjusted for practice participation in NDA; diabetes prevalence estimated using UKHLS data for Wave 7 (2015-17); **previous level refers to row above (as denominator), prevalence refers to as % of estimated total adjusted population; ^prevalence estimate for urban with significant rural based on weighted mean of prevalence estimates for urban/rural respondents to the UKHLS (no directly comparable group in UKHLS), weights based on assumption of RUC category mid-point (37.5%) of population being rurally located

For peer review only - http://bmjopen.bmj.com/site/about/guidelines.xhtml

Supplementary Table S5: Descriptive Statistics for CCGs (including "urban with significant rural" CCGs)

		Predominantly Urban CCGs (N=121)		Predominantly Rural CCGs (N=32)		Predominantly Rural CCGs (N=32)	
**Population size and age/sex structure**							
**Variable**		Mean	SD	Mean	SD	Mean	SD
Population size		283,640	166,226	285,284	178,929	311,014	223,137
% Under 40		52.85%	6.47%	43.64%	3.34%	45.15%	2.91%
% 40-64		31.25%	2.78%	33.84%	1.18%	33.43%	1.26%
% 65-79		11.62%	2.95%	16.56%	2.00%	15.66%	1.73%
% 80+		4.28%	1.16%	5.97%	0.74%	5.76%	0.91%
% female		50.49%	0.86%	50.72%	0.51%	49.05%	0.42%
**Diabetes prevalence, registrations, appropriate care and treatment targets**							
**Variable**		Mean		Mean		Mean	
NDA participation of general practices		95.84%		93.83%		92.67%	
Participation adjusted population size^		271,716		270,914		288,217	
Estimated prevalence*	Total (in adj. population)	21,206 [19,847; 22,566]		19,665 [17,115; 22,098]		21,876	
	% of population	7.80% [7.30%; 8.31%]		7.24% [6.32%; 8.16%]		7.59%	
Registrations	Total	14,750		14,089		14,932	
	% of est. prevalence	69.55%		71.64%		68.26%	
Appropriate Care	Total	6,763		6,948		7,190	
	% of est. prevalence	31.89%		35.33%		32.87%	
	% of registrations	45.85%		49.32%		48.15%	
Treatment targets	Total	5,267		4,934		5,151	
	% of est. prevalence	24.84%		25.09%		23.55%	
	% of registrations	35.71%		35.02%		34.50%	
	% of appropriate care	77.88%		71.01%		71.65%	

Notes: *prevalence data estimated from UKHLS Wave 7 (2015-2017), 95% CI in parentheses; population and rurality data from ONS; registrations, quality and treatment from NDA

For peer review only - http://bmjopen.bmj.com/site/about/guidelines.xhtml

Supplementary Table S6: Care quality, treatment target achievement and practice-level indicators of need and supply in urban and rural areas in England 2016-17 (including "urban with significant rural" CCGs)

	Predominantly Urban (CCGs=121)	Predominantly Rural (CCGs=32)	Urban with Significant Rural (CCGs=38)
Number of General Practices	4,011	902	1131
**Mean Care Quality and Treatment Target Achievement**			
% receiving all care processes	46.02%	49.39%	48.29%
% achieving all three treatment targets	35.76%	35.01%	34.58%
**Mean characteristics of registered diabetes and wider practice population**			
Total patients (list size)	7,603	8,931	8,815
Total registered diabetics	576	674	656
Practice prevalence of diabetes (%)	7.57%	7.55%	7.44%
% registered diabetics male	55.50%	56.71%	56.56%
% registered diabetics under 40	7.61%	5.94%	6.14%
% registered diabetics aged 40-64	45.11%	37.72%	37.98%
% registered diabetics aged 65-79	34.43%	40.17%	39.62%
% registered diabetics aged 80+	12.10%	15.47%	15.51%
% registered diabetics from most deprived 40% of neighbourhoods	54.20%	31.09%	31.45%
% of diabetics with ethnicity unknown	13.01%	23.05%	23.51%
% of diabetics with known ethnicity = white	70.54%	94.99%	72.04%
% of diabetics with known ethnicity = minority	29.46%	5.01%	5.09%
**Mean practice measures of labour supply**			
GPs per 1,000 patients	0.68	0.75	0.69
Nurses per 1,000 patients	0.39	0.51	0.46
Administrative staff per 1,000 patients	1.63	1.81	1.74

Notes: data on diabetes taken from the NDA; data on practice list size and labour supply from GP workforce data from NHS England; data reflect those practices on which complete data exist from both sources

For peer review only - http://bmjopen.bmj.com/site/about/guidelines.xhtml

Supplementary Table S7: Regression estimates - analyses of the % of patients receiving appropriate diabetes care quality in general practices

	I	II	III
**Urban / Rural status (reference = predominantly rural)**			
Predominantly Urban = 1	-3.370***	-1.332	-0.914
Urban with sig. rural = 1	-1.106	-0.9	-0.776
**Practice diabetic population characteristics**			
Registered diabetics (< 250) = 1		-3.250***	-3.532***
Registered diabetics (500-749) = 1		1.698*	1.887**
Registered diabetics (750 +) = 1		2.427*	2.556*
% of diabetics aged under 40		0.264*	0.284*
% of diabetics aged 40 - 64		-0.09	-0.087
% of diabetics aged 65 - 79		0.231*	0.244*
% of diabetics = male		0.003	0.018
% of patients from most deprived 40% neighbourhoods		0.034**	0.033**
% of patients from ethnic minorities		-0.067***	-0.065***
% of patients of unknown ethnicity		-0.057***	-0.057***
List size (1,000s)		-0.02	-0.479***
**Practice labour supply**			
GPs per 1000 patients			0.443***
Nurses per 1000 patients			0.330*
Admin staff per 1000 patients			0.019
Constant	49.392***	45.199***	45.831***
R-sq.	0.005	0.042	0.048
N	6,044	6,044	6,044

* p<0.05; ** p<0.01; *** p<0.001; Model I = Urban indicator only; Model II = I + Practice population characteristics; Model III = II + Practice labour supply; Models II and III include indicators for the age structure of practice list (by sex); all models estimated at practice-level using OLS weighted by the number of registered diabetics; all models clustered by general practice

For peer review only - http://bmjopen.bmj.com/site/about/guidelines.xhtml

Supplementary Table S8: Regression estimates - analyses of the % of (registered) diabetic patients achieving diabetes treatment targets in general practices (including "urban with significant rural" CCGs)

	I	II	III	IV
**Urban / rural status (reference = predominantly rural)**				
Predominantly Urban = 1	0.755**	1.062***	1.259***	1.221***
Urban with sig. rural = 1	-0.43	-0.329	-0.267	-0.279
**Appropriate care quality**				
% of patients receiving appropriate care		0.091***	0.091***	0.091***
**Practice diabetic population characteristics**				
Registered diabetics (< 250) = 1			1.552***	1.582***
Registered diabetics (500-749) = 1			-0.562*	-0.583*
Registered diabetics (750 +) = 1			-0.317	-0.33
% of diabetics aged under 40			-0.270***	-0.272***
% of diabetics aged 40 - 64			0.032	0.032
% of diabetics aged 65 - 79			0.259***	0.258***
% of diabetics = male			-0.028	-0.029
% of patients from most deprived 40% neighbourhoods			-0.010*	-0.010*
% of patients from ethnic minorities			0.030***	0.030***
% of patients of unknown ethnicity			-0.026***	-0.026***
List size (1,000s)			-0.098**	-0.055
**Practice labour supply**				
GPs per 1000 patients				-0.036
Nurses per 1000 patients				-0.029
Admin staff per 1000 patients				-0.005
Constant	35.005***	30.505***	37.728***	4.872
R-sq.	0.005	0.062	0.139	0.139
N	6,044	6,044	6,044	6,044

* p<0.05; ** p<0.01; *** p<0.001; Model I = Urban indicator only; Model II = I + Care quality; Model III = II + Practice population characteristics; Model IV = III + Practice labour supply; Models III + IV include indicators for the age structure of practice list (by sex); all models estimated at practice-level using OLS weighted by the number of registered diabetics; all models clustered by general practice

For peer review only - http://bmjopen.bmj.com/site/about/guidelines.xhtml

[EMBED SUPPLEMENTARY FIGURE S1 HERE]

For peer review only

For peer review only - http://bmjopen.bmj.com/site/about/guidelines.xhtml

Reporting checklist for cross sectional study.

Based on the STROBE cross sectional guidelines.

Instructions to authors

Complete this checklist by entering the page numbers from your manuscript where readers will find each of the items listed below.

Your article may not currently address all the items on the checklist. Please modify your text to include the missing information. If you are certain that an item does not apply, please write "n/a" and provide a short explanation.

Upload your completed checklist as an extra file when you submit to a journal.

In your methods section, say that you used the STROBE cross sectionalreporting guidelines, and cite them as:

von Elm E, Altman DG, Egger M, Pocock SJ, Gotzsche PC, Vandenbroucke JP. The Strengthening the Reporting of Observational Studies in Epidemiology (STROBE) Statement: guidelines for reporting observational studies.

		Reporting Item	Page Number
**Title and abstract**			
Title	#1a	Indicate the study's design with a commonly used term in the title or the abstract	1

For peer review only

Abstract	#1b	Provide in the abstract an informative and balanced summary of what was done and what was found	1, 2
**Introduction**			
Background / rationale	#2	Explain the scientific background and rationale for the investigation being reported	3, 4
Objectives	#3	State specific objectives, including any prespecified hypotheses	4
**Methods**			
Study design	#4	Present key elements of study design early in the paper	7, 8, 9
Setting	#5	Describe the setting, locations, and relevant dates, including periods of recruitment, exposure, follow-up, and data collection	5, 6
Eligibility criteria	#6a	Give the eligibility criteria, and the sources and methods of selection of participants.	n/a
	#7	Clearly define all outcomes, exposures, predictors, potential confounders, and effect modifiers. Give diagnostic criteria, if applicable	7, 8, 9
Data sources / measurement	#8	For each variable of interest give sources of data and details of methods of assessment (measurement). Describe comparability of assessment methods if there is more than one group. Give information separately for for exposed and unexposed groups if applicable.	4, 5, 6

For peer review only

Bias	#9	Describe any efforts to address potential sources of bias	7, 8, 9, 16
Study size	#10	Explain how the study size was arrived at	7, 8, 9
Quantitative variables	#11	Explain how quantitative variables were handled in the analyses. If applicable, describe which groupings were chosen, and why	7, 8, 9
Statistical methods	#12a	Describe all statistical methods, including those used to control for confounding	7, 8, 9
Statistical methods	#12b	Describe any methods used to examine subgroups and interactions	7, 8, 9
Statistical methods	#12c	Explain how missing data were addressed	n/a
Statistical methods	#12d	If applicable, describe analytical methods taking account of sampling strategy	n/a
Statistical methods	#12e	Describe any sensitivity analyses	n/a
**Results**			
Participants	#13a	Report numbers of individuals at each stage of study—eg numbers potentially eligible, examined for eligibility, confirmed eligible, included in the study, completing follow-up, and analysed. Give information separately for for exposed and unexposed groups if applicable.	22 - 26

For peer review only

Participants	#13b	Give reasons for non-participation at each stage	n/a
Participants	#13c	Consider use of a flow diagram	n/a
Descriptive data	#14a	Give characteristics of study participants (eg demographic, clinical, social) and information on exposures and potential confounders. Give information separately for exposed and unexposed groups if applicable.	22 - 26
Descriptive data	#14b	Indicate number of participants with missing data for each variable of interest	n/a
Outcome data	#15	Report numbers of outcome events or summary measures. Give information separately for exposed and unexposed groups if applicable.	n/a
Main results	#16a	Give unadjusted estimates and, if applicable, confounder-adjusted estimates and their precision (eg, 95% confidence interval). Make clear which confounders were adjusted for and why they were included	13, 14, 22, 26
Main results	#16b	Report category boundaries when continuous variables were categorized	13, 14, 24, 26
Main results	#16c	If relevant, consider translating estimates of relative risk into absolute risk for a meaningful time period	n/a
Other analyses	#17	Report other analyses done—e.g., analyses of subgroups and interactions, and sensitivity analyses	22-26

Discussion

For peer review only

Key results	#18	Summarise key results with reference to study objectives	15
Limitations	#19	Discuss limitations of the study, taking into account sources of potential bias or imprecision. Discuss both direction and magnitude of any potential bias.	16, 17
Interpretation	#20	Give a cautious overall interpretation considering objectives, limitations, multiplicity of analyses, results from similar studies, and other relevant evidence.	15, 16
Generalisability	#21	Discuss the generalisability (external validity) of the study results	16, 17
**Other Information**			
Funding	#22	Give the source of funding and the role of the funders for the present study and, if applicable, for the original study on which the present article is based	1, 2

None The STROBE checklist is distributed under the terms of the Creative Commons Attribution License CC-BY. This checklist can be completed online using https://www.goodreports.org/, a tool made by the EQUATOR Network in collaboration with Penelope.ai

For peer review only
